# Transcriptomic profiling revealed key signaling pathways for cold tolerance and acclimation of two carp species

**DOI:** 10.1186/s12864-020-06946-8

**Published:** 2020-08-05

**Authors:** Guodong Ge, Yong Long, Lianyu Shi, Jing Ren, Junjun Yan, Chitao Li, Qing Li, Zongbin Cui

**Affiliations:** 1 State Key Laboratory of Freshwater Ecology and Biotechnology, Institute of Hydrobiology, Chinese Academy of Sciences, Wuhan, 430072 China; 2 University of Chinese Academy of Sciences, Beijing, 100049 China; 3 Heilongjiang River Fishery Research Institute of Chinese Academy of Fishery Sciences, Ha’erbin, 150070 China; 4 State Key Laboratory of Applied Microbiology Southern China, Guangdong Institute of Microbiology, Guangdong Academy of Sciences, Guangzhou, 510070 China

**Keywords:** Carp, Cold tolerance, Cold acclimation, RNA-Seq, Biological process, Signaling pathways

## Abstract

**Background:**

Closely related species of the carp family (*Cyprinidae*) have evolved distinctive abilities to survive under cold stress, but molecular mechanisms underlying the generation of cold resistance remain largely unknown. In this study, we compared transcriptomic profiles of two carp species to identify key factors and pathways for cold tolerance and acclimation.

**Results:**

Larvae of Songpu mirror carp and Barbless carp that were pretreated at 18 °C for 24 h significantly improved their survival rates under lethal cold temperature at 8 °C or 10 °C, indicating that two carp species possess the ability of cold acclimation. However, Songpu mirror carp exhibited stronger abilities of cold tolerance and acclimation than Barbless carp. Transcriptomic profiles of Songpu mirror carp and Barbless carp larvae at 28 °C and 18 °C were compared during cold acclimation through RNA-seq. Differentially expressed genes that are closely associated with the differences in cold acclimation between two carp species were identified through bioinformatics and Venn’s diagram analysis. GO enrichment analysis of these genes indicated that cellular component assembly involved in morphogenesis, secondary alcohol metabolism and drug transport were the most up-regulated biological processes during cold acclimation of Songpu mirror carp. Conversely, positive regulation of macroautophagy, intracellular protein transport, and organonitrogen compound catabolism were the most down-regulated biological processes during cold acclimation of Barbless carp. KEGG enrichment analysis revealed that factors in the FoxO-related signaling pathways are mainly responsible for the development of differences in cold tolerance and acclimation between two carp species since altering the phosphorylation of key proteins in the FoxO-related signaling pathways with inhibitors or an activator significantly decreased the cold tolerance and acclimation of Songpu mirror carp. These data provided key clues for dissection of molecular mechanisms underlying the development of cold tolerance and acclimation in carps.

**Conclusions:**

These findings indicate that larvae of two carp species possess different abilities of cold tolerance and can build cold acclimation under mild low temperature. Multiple biological processes and FoxO-related signaling pathways are closely associated with the development of differences in cold tolerance and acclimation between two carp species.

## Background

Temperature is a master abiotic factor that can affect almost all life activities of fishes, including growth, development, reproduction, metabolism, behavior and geographical distribution [1]. Low temperature beyond the temperature windows of fish tolerance often becomes one of natural disasters for fish farming [2, 3]. To cope with a sharp decrease in water temperature, different fish species have evolved various abilities for survival. Many fish species can endure the adverse effects of low temperature by producing different types of muscle fibers and remodeling the tissue structure of organs such as gills and heart [4–6]. Fish living in the Arctic and Antarctic regions have evolved a variety of biochemical and physiological mechanisms, including synthesis of antifreeze proteins (AFP) [7], antifreeze glycoproteins (AFGP) [8, 9] and tubulin [10], and lack of hemoglobin [11] to survive in low temperature environments. Antarctic notothenioids have evolved to become both cold adapted and cold specialized and a marked signature of cold specialization is an apparent loss of the cellular heat shock response (HSR) [12]. The transcriptional response to acute heat stress was minimal in cold-adapted and red-blooded icefish *P. borchgrevinki*, but robust responses in the *C. rastrospinosus* that represents the hemoglobinless Antarctic notothenioids occurred in the broader cellular networks especially in inflammatory responses despite lacking the classic HSR and unfolded protein response (UPR) [13]. In addition, many of closely related fish species including some members of the carp family exhibit distinctive abilities of tolerance to low temperature [14].

Effects of cold stress on fish cells and individuals were well documented during the past decades. Previous studies have shown that the rate of protein synthesis, the activity of enzymes, the respiration rate and oxygen consumption in the cell became slower and the number of mitochondria decreased under low temperature [15–17]. Low temperature can also alter the permeability of biological membranes, decrease the membrane fluidity as the result of stabilizing weak chemical bonds, and affect functional properties of the proteins within the membranes [18]. Effects of acute exposure to low temperature on cellular membranes include alterations in membrane phospholipid composition, membrane phase behavior and membrane thickness [19]. The increase of the bilayer thickness can cause the activation of some enzymes and the expression of some genes associated with cold resistance [20–24]. Cold stress depressed phagocytic activity and antibody levels in tilapia (*Oreochromis aureus*) by modulating the changes of catecholamines and cortisol [25]. In addition, thyroxine can affect the functions of muscles, heart and other organs by regulating metabolism, enzyme activity, and gene expression and thus participates the resistance to low-temperature stress of zebrafish [26, 27]. In adult zebrafish, autophagy and lipid catabolism play an important role in improving zebrafish survival rate to acute cold stress [28, 29]. We have recently found that cold exposure can cause apoptosis in zebrafish larvae through suppression of sarcoplasmic/ER Ca^2+^-ATPase (SERCA) activity and induction of unfolded protein response (UPR) and ER stress [30]. These findings have provided crucial clues for understanding the biochemical and molecular mechanisms of self-protection in fish under low temperature.

Many of fish species were found to have the acclimation abilities to cold stress and an increasing numbers of factors were identified to protect fish body from cold injury during cold acclimation. Growth hormone is reported to involve in the process of temperature acclimatization by suppressing the synthesis of AFP [31]. SERCA is associated with muscle and heart function in cold water and thyroxine treatment restored heart rate and SERCA activity in hypothyroid fish [27, 32], indicating that thyroxine plays an important role in the maintenance of heart function during cold acclimation. We have previously found that many genes and alternative splicing were affected during cold acclimation of zebrafish larvae [33, 34]. It is suggested that organisms react to the changes of environmental temperature stimuli via the perception of signals and subsequent alterations of gene expression [35]. However, little is known about how fish senses cold signal and what signaling pathways in fish cells are regulated during cold acclimation.

Members of the carp family have shown differential abilities of tolerance to low temperature [36], but the molecular basis underlying the difference in cold tolerance remains poorly understood. Previous studies have reported that Songpu mirror carp (*Cyprinus carpio.*) have a strong ability of cold tolerance [37, 38] and Barbless carp (*Cyprinus pellegrini*) that specifically live in Xingyun Lake of the Yunnan-Guizhou Plateau in China [39] are sensitive to cold temperature [36]. In this study, two of closely related members of the carp family, Songpu mirror carp and Barbless carp that can hybrid to generate the offspring [39], were used to identify differentially expressed factors and activated signaling pathways that may play crucial roles in the development of differences in cold tolerance and acclimation of carps.

## Results

### Cold tolerance and acclimation of Songpu mirror carp and barbless carp

**Fig. 1:**
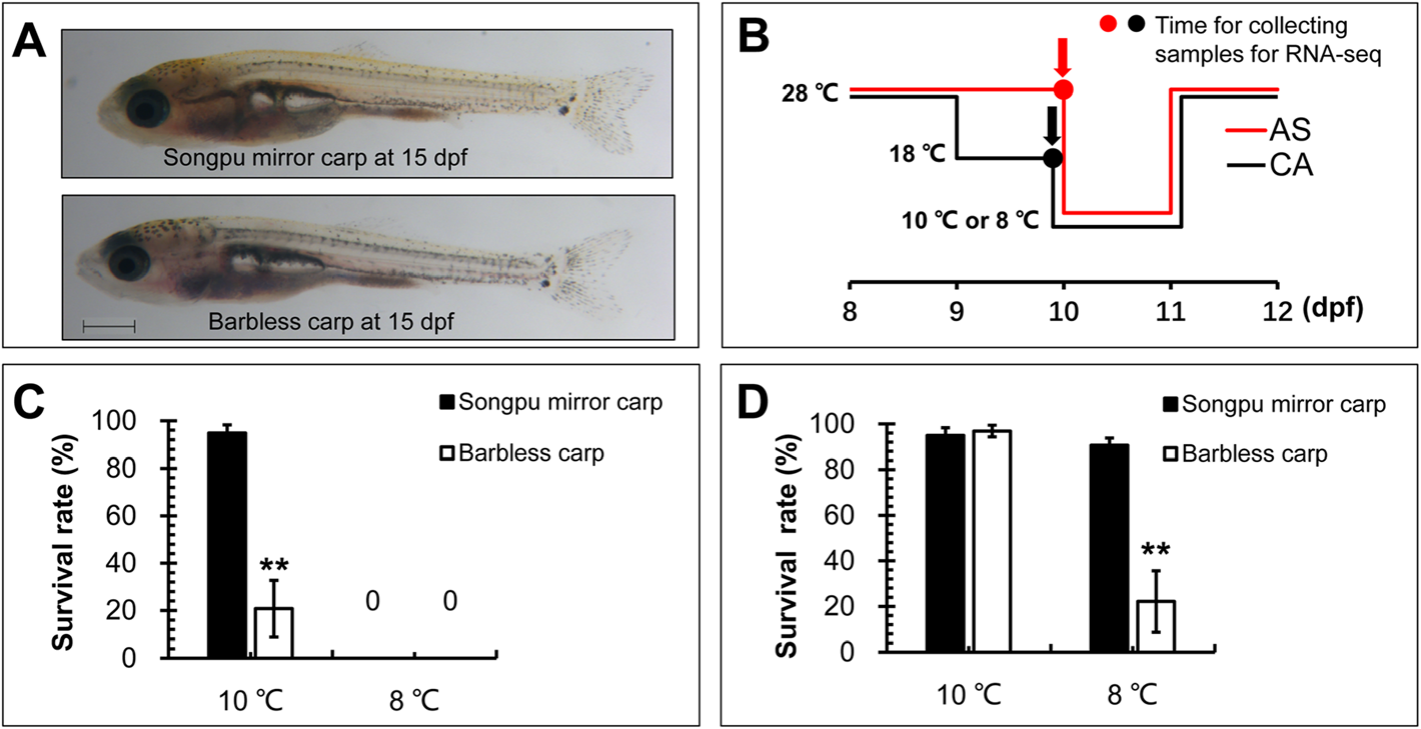
The difference of Songpu mirror carp and Barbless carp in cold tolerance and cold acclimation. **a** Lateral views of Songpu mirror carp (upper) and Barbless carp (below) at 15 dpf. **b** Schematic presentation of the experiments for cold tolerance (acute stress in red line, AS) and cold acclimation (CA in black). Carp larvae at 9 dpf in AS group were directly transferred from normal rearing temperature (28 °C) to lethal cold stress (10 °C) or extreme lethal cold stress (8 °C) for 24 h. Carp larvae at 9 dpf in CA group were transferred from normal rearing temperature (28 °C) to mild low temperature (18 °C) for 24 h followed by exposure to lethal cold (10 °C) or extreme lethal cold (8 °C) for 24 h. Then, cold-treated larvae were recovered at 28 °C for 24 h and survival rates of larvae in AS and CA groups were calculated at 12 dpf. Larvae in AS and CA groups were quickly sampled at 10 dpf (pointed by red and black arrows) for total RNA isolation and served as the control (Ctrl) and cold acclimation (CA) groups of RNA-seq analysis, respectively. **c** Survival rates of Songpu mirror carp and Barbless carp at 12 dpf in AS groups. **d** Survival rates of Songpu mirror carp and Barbless carp at 12 dpf in CA groups. **, *p* < 0.01

Songpu mirror carp and Barbless carp are two closely related species whose larvae rearing at 28 °C exhibited similar developmental phenotypes at 15 dpf (Fig. 1a). Larvae of Songpu mirror carp and Barbless carp at 9 dpf were used to compare their differences in tolerance to lethal cold stress at 10 °C or 8 °C for 24 h (AS) and in cold acclimation to mild cold stress at 18 °C for 24 h followed by exposure to lethal cold stress at 10 °C or 8 °C for 24 h (CA) (Fig. 1b). Then, larvae in AS and CA groups were recovered at 28 °C for another 24 h to monitor their survival rates.

As shown in Fig. 1c, the survival rate of Songpu mirror carp at 12 dpf was significantly higher than that of Barbless carp after exposure to lethal cold stress at 10 °C, while no living larvae were detected in AS groups of Songpu mirror carp and Barbless carp exposed to lethal cold stress at 8 °C. These data indicate that Songpu mirror carp has a stronger ability of cold tolerance to lethal cold stress than Barbless carp.

We further compared the difference of Songpu mirror carp and Barbless carp in cold acclimation. After pretreatment of 9 dpf larvae at 18 °C for 24 h, 94.91% of Songpu mirror carp larvae and 96.84% of Barbless carp larvae in CA groups were able to survival at lethal cold stress at 10 °C, while 90.61% of Songpu mirror larvae and 22.12% of Barbless carp larvae in CA group were able to survival at lethal cold stress at 8 °C (Fig. 1d) (Additional file 1), indicating that both Songpu mirror carp and Barbless carp possess the ability of cold acclimation and Songpu mirror carp has a stronger ability of cold acclimation than Barbless carp.

### RNA-seq and bioinformatics analysis

**Fig. 2:**
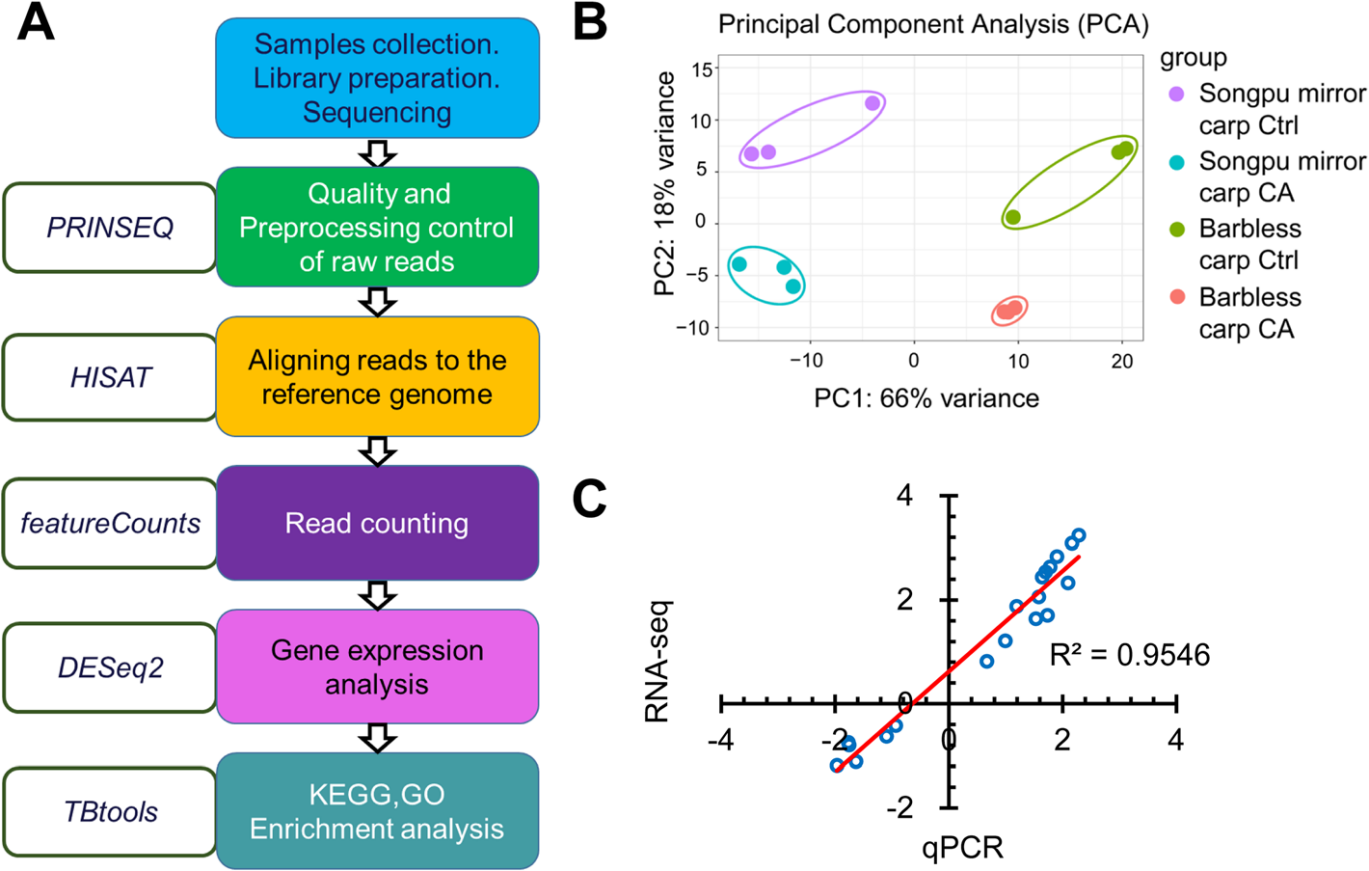
The workflow of RNA-seq data analysis. **a** Main steps and bioinformatics tools used in the study. The programs for data analysis were shown in colored boxes and the software packages in transparent boxes. **b** Principal component analysis (PCA) of gene expression profiles for four different groups. The distance between the groups indicates the variance. The colored dots represent data replicates within the same group: purple for the control (Ctrl) of Songpu mirror carp, blue for the cold acclimation (CA) of Songpu mirror carp, green for the control (Ctrl) of Barbless carp, red for the cold acclimation (CA) of Barbless carp. **c** Validation of RNA-seq data using qPCR. Log2 Fold changes of RNA-seq data for gene expression were plotted against those of qPCR data. The reference line in red indicates the linear relationship between the results of RNA-Seq and qPCR

A total of twelve cDNA libraries for Songpu mirror carp and Barbless carp were constructed and subjected to high-throughput sequencing, followed by extensive bioinformatics analysis (Fig. 2a). The total number of raw read pairs ranged from 11.93 to 15.26 million (M) and about 60% of the processed reads were mapped to the reference genome of common carp [40] after filtering out low quality reads (Table 1).

**Table 1:** Statistics for the mapping of reads

	Songpu mirror carp						Barbless carp					
	Ctrl_1	Ctrl_2	Ctrl_3	CA_1	CA_2	CA_3	Ctrl_1	Ctrl_2	Ctrl_3	CA_1	CA_2	CA_3
Total reads(M)	12.05X2	11.93X2	13.14X2	12.88X2	12.62X2	13.26X2	15.26X2	14.12X2	14.49X2	13.34X2	13.82X2	13.82X2
Good reads(M)	18.96	16.78	17.78	16.74	22.55	22.74	25.25	20.60	24.07	22.25	24.79	20.38
% Good reads(M)	78.68	70.37	67.64	64.99	89.35	85.72	82.70	72.94	83.07	83.38	89.69	73.72
Overall alignment rate (%)	65.7	68.23	53.87	61.72	67.69	63.15	65.23	65.74	64.81	68.16	63.92	66.98

Principle component analysis (PCA) was used to examine the transcription profiles of genes in AS and CA groups. As shown in Fig. 2b, there is an obvious consistency within the same group and a clear discrepancy among groups in PCA results, suggesting small variations within the same group and a high reliability of data. Moreover, the influence of cold stress at 18 °C led to 18% of the transcriptional variations (PC2) and 66% of variations (PC1) was due to the difference of two carp species (Fig. 2b), indicating that Songpu mirror carp and Barbless carp are two closely related species of the carp family that possess different abilities of cold acclimation.

To validate the expression profiles from RNA-seq analysis, 13 genes including two transcripts of *hmgb1* were measured with qPCR assays. Primers used for qPCR were listed in Additional file 2. The subunit S11 of the ribosomal gene 40S was used as the reference gene. As shown in Fig. 2c and Additional file 3, the Spearman bivariate correlation analysis revealed that data of RNA-seq and qPCR were significantly correlated (*p* < 0.00001, correlation coefficient = 0.9546), indicating the reliability of RNA-seq data.

### Identification of differentially expressed genes for cold tolerance and acclimation between Songpu mirror carp and barbless carp

**Fig. 3:**
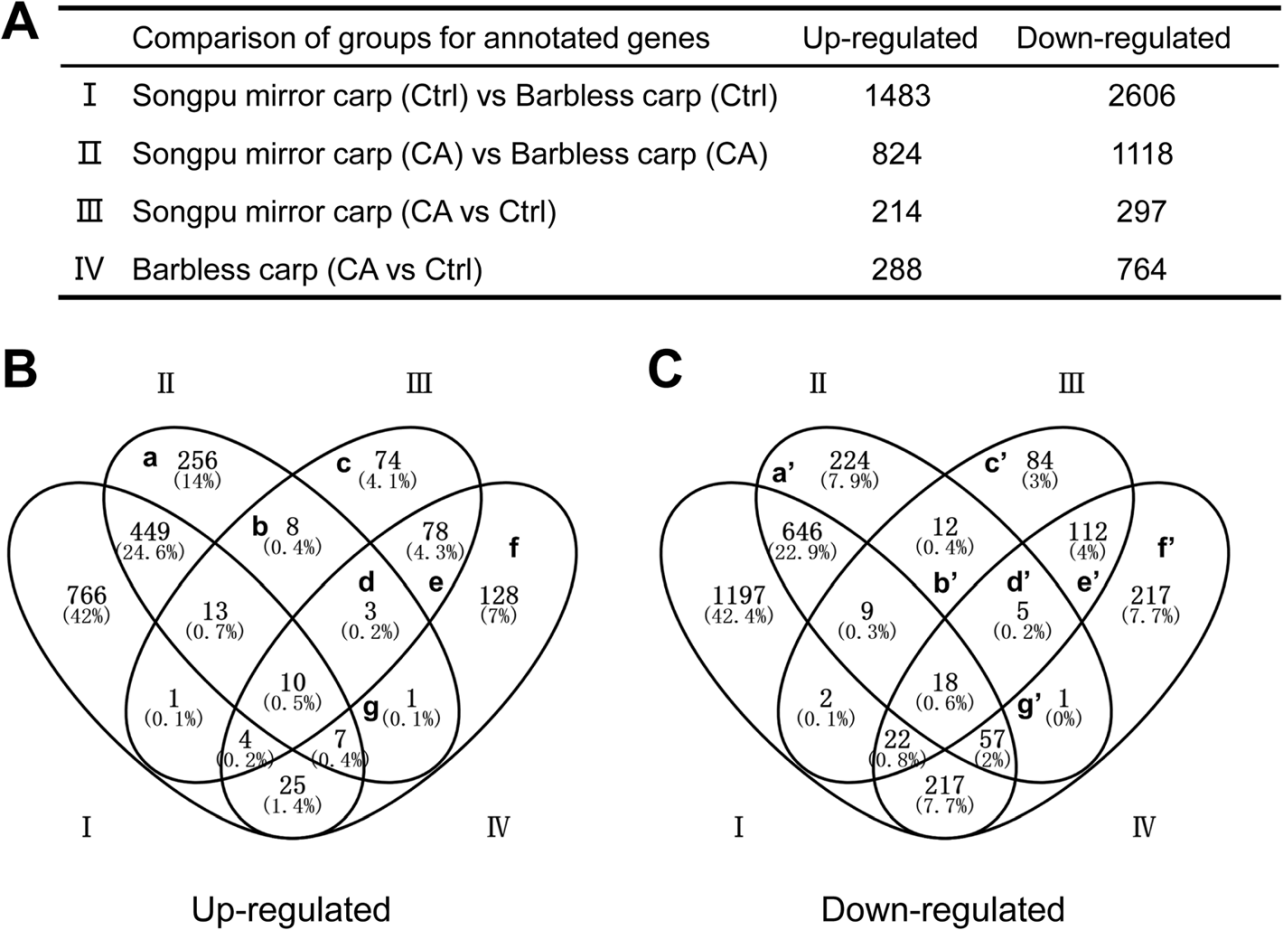
Identify genes involved in cold acclimation of Songpu mirror carp and Barbless carp. **(A)** The number of differentially expressed genes between two groups was shown in Roman numerals. Genes involved in cold acclimation of Songpu mirror carp and Barbless carp were identified through Venn’s diagrams analysis of up-regulated genes **(B)** and down-regulated genes **(C).** Differentially expressed genes in group I represent the difference of two carp species in gene expression under normal rearing temperature at 28 °C. Differentially expressed genes in groups II and III (a, b, c, a’, b’ and c’) encode specific factors for the formation of cold acclimation in Songpu mirror carp, and those in group IV (f, g, f’ and g’) encode specific factors for the formation of cold acclimation in Barbless carp. Common factors required for the formation of cold acclimation of two carp species are encoded by differentially expressed genes that are overlapped in group III and IV (d, e, d’ and e’)

After obtaining counts for annotated genes in two control groups (Ctrl) and cold acclimation groups (CA) of Songpu mirror carp and Barbless carp, we performed a comparative analysis of differentially expressed genes between two of Ctrl and/or CA groups (Fig. 3a). Differentially expressed genes in group I represent the developmental and physiological differences between Songpu mirror carp and Barbless carp growing at normal temperature. Differentially expressed genes in group II represent the differences between Songpu mirror carp and Barbless carp during cold acclimation. Differentially expressed genes in group III represent cold-induced and -inhibited genes in Songpu mirror carp. Differentially expressed genes in group IV represent cold-induced and -inhibited genes in Barbless carp. The detail information of genes in each groups were listed in Additional file 4.

Next, we performed analysis of differentially genes with Venn’s diagrams to identify potential genes that are specifically required for cold tolerance of Songpu mirror carp and for cold acclimation of Songpu mirror carp and Barbless carp. As shown in Fig. 3b and c, differentially expressed genes in group I contain genes that are responsible for the difference of Songpu mirror carp and Barbless carp in cold tolerance to lethal stress (Fig. 1c). Some of differentially expressed genes in groups II and III (a, b, c, a’, b’, and c’) contain candidate genes that are responsible for the strong ability of cold acclimation in Songpu mirror carp. Some of differentially expressed genes in groups III and IV (d, e, d’ and e’) are involved in cold acclimation of both Songpu mirror carp and Barbless carp. Some of differentially expressed genes in group IV (f, g, f’ and g’) are likely required for cold acclimation of Barbless carp. The detail information and fold change of genes in each sets obtained by Venn’s diagrams were displayed in Additional file 5. These sets of differentially expressed genes were further used for enrichment analysis to explore biological processes and signaling pathways for cold acclimation.

### GO enrichment analysis to identify biological processes for the difference in cold acclimation between Songpu mirror carp and barbless carp

**Fig. 4:**
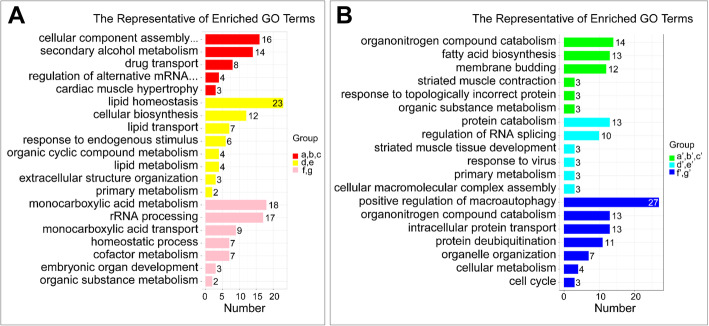
Representative terms of GO enrichment analysis**.** GO term redundancy was reduced by REVIGO tool to give a representative subset of terms in different groups of up-regulated genes **(A)** and down-regulated genes **(B)**. Representative biological processes of different groups are shown in different colors

Differentially expressed genes that are potentially associated with cold acclimation of Songpu mirror carp and Barbless carp were analyzed with GO enrichment and displayed in Additional file 6 and representatives of GO term belonged to the biological process obtained through REVIGO tool were displayed in Additional file 7. Candidate genes up-regulated in groups II and III (a, b, c) during the formation of a strong cold acclimation in Songpu mirror carp were overrepresented in biological processes including cellular component assembly involved in morphogenesis, secondary alcohol metabolism, drug transport, regulation of alternative mRNA splicing via spliceosome, cardiac muscle hypertrophy and cell-substrate adhesion (Fig. 4a) and details of these processes can be visualized with the REVIGO Web server in Figure S1. Candidate genes down-regulated in groups II and III (a’, b’ and c’) during the formation of strong cold acclimation in Songpu mirror carp were mainly enriched in biological processes including organonitrogen compound catabolism, fatty acid biosynthesis, membrane budding, striated muscle contraction, response to topologically incorrect protein and organic substance metabolism (Fig. 4b and S2).

Candidate genes up-regulated in groups III and IV (d and e) during cold acclimation of both Songpu mirror carp and Barbless carp were mainly enriched in biological processes including lipid homeostasis, cellular biosynthesis, lipid transport, response to endogenous stimulus and other processes (Fig. 4a and S3). Candidate genes down-regulated in groups III and IV (d’ and e’) during cold acclimation of both Songpu mirror carp and Barbless carp were overrepresented in biological processes such as protein catabolism, regulation of RNA splicing, striated muscle tissue development and primary metabolism (Fig. 4b and S4).

Candidate genes up-regulated in groups III and IV (f and g) during cold acclimation of Barbless carp were mainly enriched in biological processes including monocarboxylic acid transport, rRNA processing, and monocarboxylic acid transport, cofactor metabolism and other processes (Fig. 4a and S5). Candidate genes down-regulated in groups III and IV (f’ and g’) during cold acclimation of Barbless carp were overrepresented in biological processes including positive regulation of macroautophagy, organonitrogen compound catabolism, intracellular protein transport, protein deubiquitination and other processes (Fig. 4b and S6).

### KEGG enrichment analysis to identify signaling pathways for cold acclimation difference between Songpu mirror carp and barbless carp

**Fig. 5:**
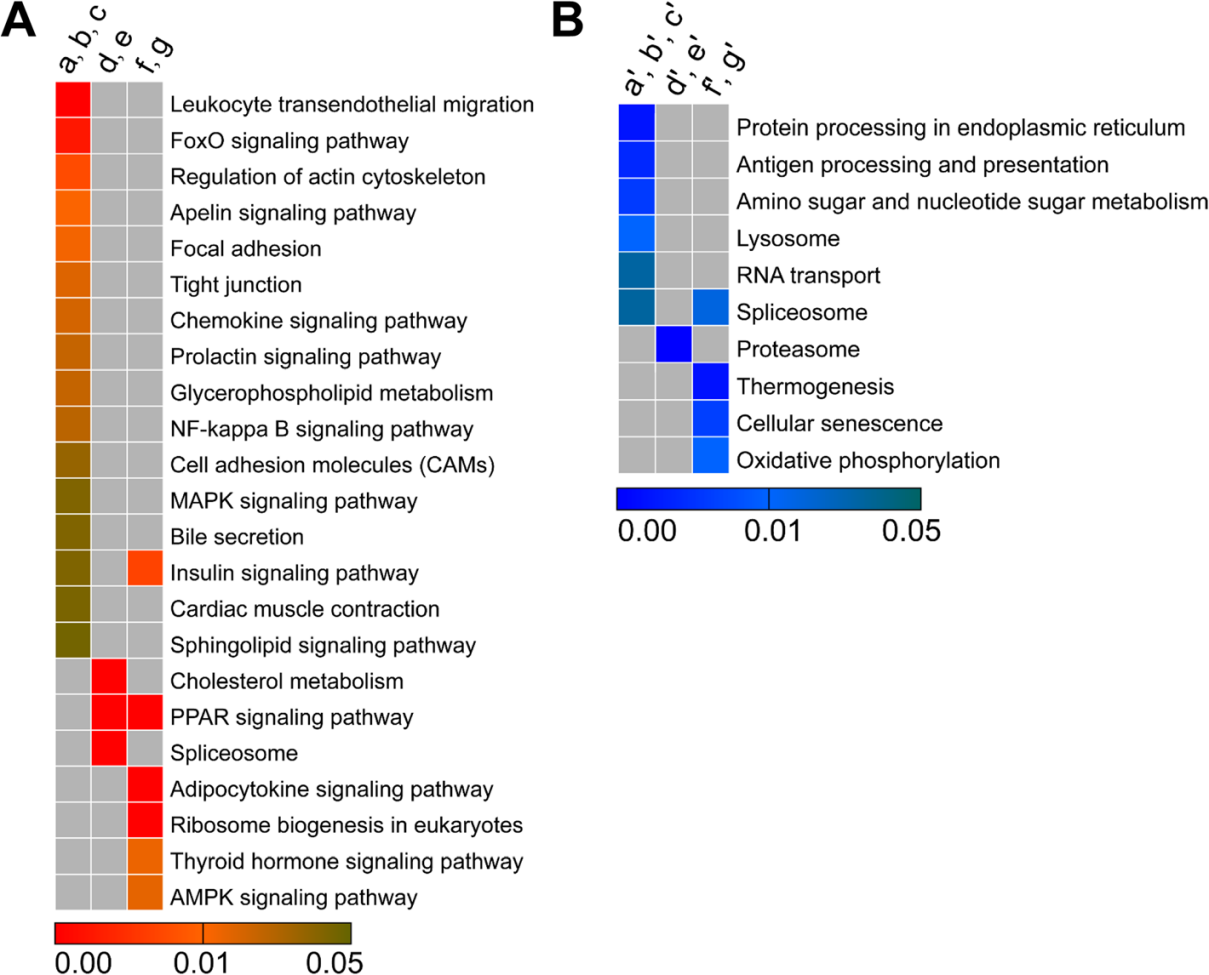
Heat maps from KEGG enrichment analysis of genes associated with cold acclimation in Songpu mirror carp and Barbless carp. **(A)** Up-regulated genes. **(B)** Down-regulated genes. KEGG enrichment analysis of differentially expressed genes identified by Venn’s diagram in different groups. Differentially expressed genes in groups II and III (a, b, c, a’, b’ and c’) were enriched in signaling pathways that are potentially required for the formation of cold acclimation in Songpu mirror carp. Differentially expressed genes in group IV (f, g, f’ and g’) were enriched in signaling pathways that are likely important for the formation of cold acclimation in Barbless carp. Differentially expressed genes in groups III and IV (d, e, d’ and e’) were enriched in signaling pathways that are potentially required for the formation of cold acclimation in two carp species

Differentially expressed genes that are potentially associated with cold acclimation of Songpu mirror carp and Barbless carp were analyzed with the KEGG pathway enrichment. Candidate genes up-regulated in groups II and III (a, b, c) during the formation of a strong cold acclimation in Songpu mirror carp were mostly enriched in signaling pathways such as leukocyte transendothelial migration, regulation of actin cytoskeleton, and FoxO signaling pathway. (Fig. 5a and Additional file 8). Candidate genes down-regulated in groups II and III (a’, b’ and c’) during the formation of strong cold acclimation in Songpu mirror carp were mainly overrepresented in signaling pathways such as protein processing in endoplasmic reticulum, antigen processing and presentation and amino sugar and nucleotide sugar metabolism (Fig. 5b and Additional file 8). These signaling pathways are mainly classified into environmental information processing (Table 2).

**Table 2:** Main classes of KEGG terms for up-regulated genes

Main Classes	Term names in different groups		
	a, b, c	d, e	f
Environmental information processing	FoxO signaling pathway; Apelin signaling pathway; NF-kappa B signaling pathway; Cell adhesion molecules; MAPK signaling pathway; Sphingolipid signaling pathway		AMPK signaling pathway
Organismal systems	Bile secretion; Insulin signaling pathway; Leukocyte transendothelial migration; Chemokine signaling pathway; Prolactin signaling pathway; Cardiac muscle contraction	PPAR signaling pathway; Cholesterol metabolism	PPAR signaling pathway; Insulin signaling pathway; Thyroid hormone signaling pathway; Adipocytokine signaling pathway
Genetic information processing		Spliceosome	Ribosome biogenesis in eukaryotes
Cellular processes	Regulation of actin cytoskeleton; Focal adhesion; Tight junction		
Metabolism	Glycerophospholipid metabolism		

Candidate genes up-regulated in groups III and IV (d and e) during cold acclimation of both Songpu mirror carp and Barbless carp were enriched in signaling pathways including cholesterol metabolism, PPAR signaling pathway and spliceosome (Fig. 5a and Additional file 8). Candidate genes down-regulated in groups III and IV (d’ and e’) during cold acclimation of both Songpu mirror carp and Barbless carp were mainly overrepresented in the signaling pathway of proteasome (Fig. 5b and Additional file 8). These signaling pathways are classified into organism systems (Table 2).

Candidate genes up-regulated in groups III and IV (f and g) during cold acclimation of Barbless carp were mainly enriched in signaling pathways such as insulin signaling pathway, PPAR signaling pathways, adipocytokine signaling pathway and ribosome biogenesis in eukaryotes (Fig. 5a and Additional file 8). Candidate genes down-regulated in groups III and IV (f’ and g’) during cold acclimation of Barbless carp were overrepresented in signaling pathways of thermogenesis, cellular senescence and oxidative phosphorylation (Fig. 5b and Additional file 8). These three signaling pathways are classified into organism systems, cellular processes, and metabolism (Table 3).

**Table 3:** Main classes of KEGG terms for down-regulated genes

Main Class	Term names in different groups		
	a’, b’, c’	d’, e’	f’
Organismal Systems	Antigen processing and presentation		Thermogenesis
Genetic information processing	Spliceosome; Protein processing in endoplasmic reticulum; RNA transport	Proteasome	Spliceosome
Cellular processes	Lysosome		Cellular senescence
Metabolism	Amino sugar and nucleotide sugar metabolism		Oxidative phosphorylation

### FoxO signaling pathway played a key role in the difference of cold acclimation between Songpu mirror carp and barbless carp

**Fig. 6:**
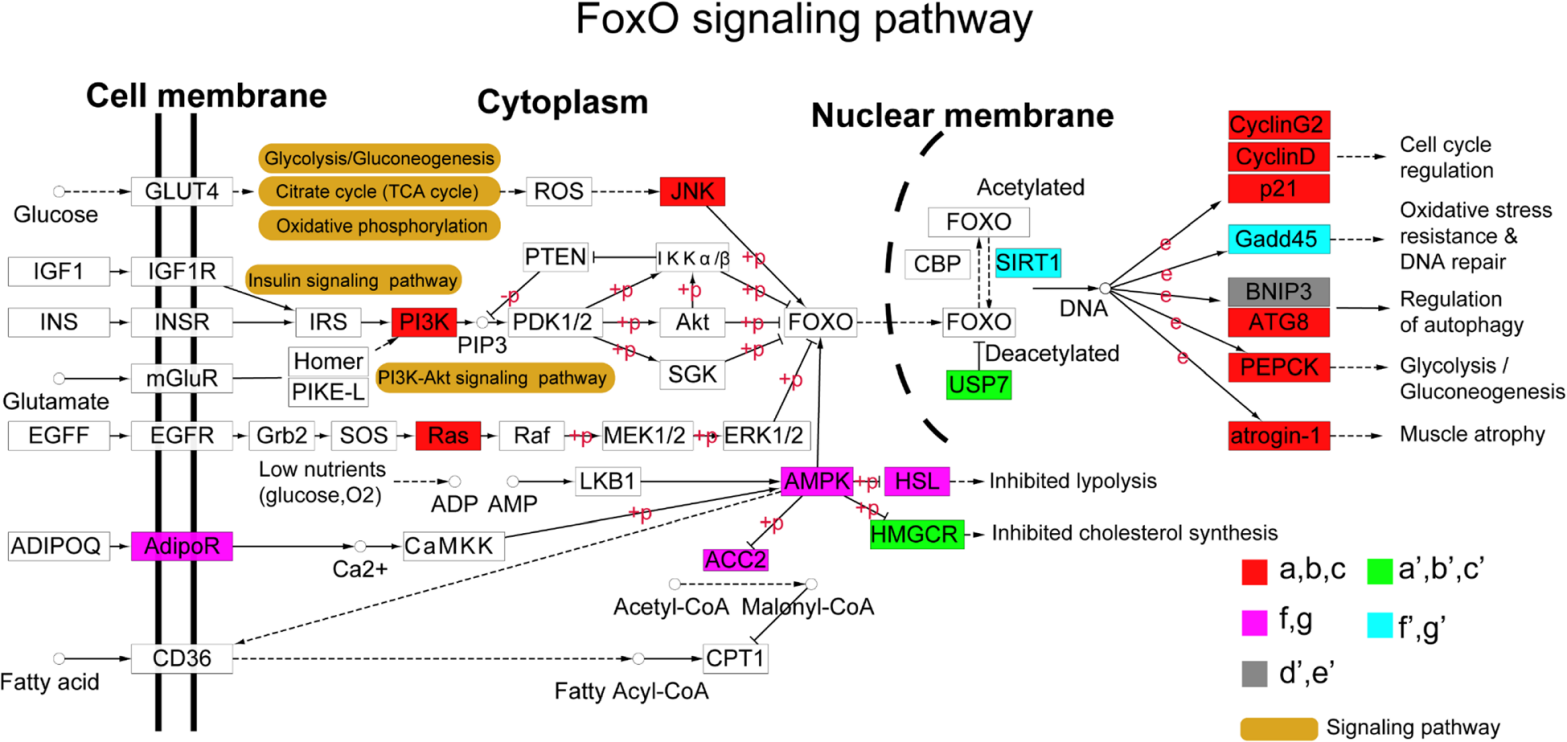
Signaling pathways associated with cold acclimation of two carp species activated in the FoxO signaling pathway. The differentially expressed genes activated in different groups were showed in colors. The –p and + p indicate dephosphorylation and phosphorylation, respectively

FOXO is a transcription factor that plays important roles in transcriptional expression of genes involved in the control of cell survival [41]. The activity of FOXO was regulated by multiply mechanisms [41]. In this study, nine of up-regulated genes (a, b and c, in red) and two of down-regulated genes (a’, b’ and c’, in green) in groups II and III during the cold acclimation of Songpu mirror carp were mapped to the FoxO signaling pathway and cellular events that were associated with FoxO signaling pathway include cell cycle regulation, regulation of autophagy, glycolysis/gluconeogenesis, muscle atrophy and cholesterol synthesis (Fig. 6 and Table 4). Regulation of autophagy was down-regulated by cold-inhibited BNIP3 (Bcl-2/adenovirus E1B 19-kDa interacting protein) expression in groups III and IV (d’ and e’ in gray) of both Songpu mirror carp and Barbless carp (Fig. 6). During the cold acclimation of Barbless carp, the AMPK signaling pathway was mainly activated by up-regulation of four genes in group IV (f and g in magenta) (Table 5) and oxidative stress resistance & DNA repair events downstream of FoxO signaling pathway were negatively controlled by down-regulation of two genes in group IV (f’ and g’ in aqua).

**Table 4:** Up-regulated folds of genes in groups (a, b and c) mapped to FoxO signaling pathways

Genes	Log2Fold	Protein names
*kras*	1.33	GTPase KRas
*ccnd2*	1.29	G1/S-specific cyclin-D2
*cdkn1a*	1.24	Cyclin-dependent kinase inhibitor 1 (p21)
*pik3r5*	1.10	Phosphoinositide 3-kinase regulatory subunit 5
*jnk1*	1.06	Stress-activated protein kinase JNK1
*ccng2*	1.00	Cyclin-G2
*hras*	0.97	GTPase HRas
*fbxo25*	0.91	F-box only protein 25
*pck1*	0.90	Phosphoenolpyruvate carboxykinase, cytosolic (PEPCK-C)
*lgg-1*	0.71	Protein lgg-1

**Table 5:** Up-regulated folds of genes in groups (f and g) mapped to AMPK signaling pathway

Genes	Log2Fold	Protein names
*pck2*	1.09	Phosphoenolpyruvate carboxykinase [GTP], mitochondrial (PEPCK-M)
*prkag2*	1.08	5′-AMP-activated protein kinase subunit gamma-2
*lipe*	0.96	Hormone-sensitive lipase
*adipor1*	0.80	Adiponectin receptor protein 1
*acc2*	0.71	Acetyl-CoA carboxylase 2

**Fig. 7:**
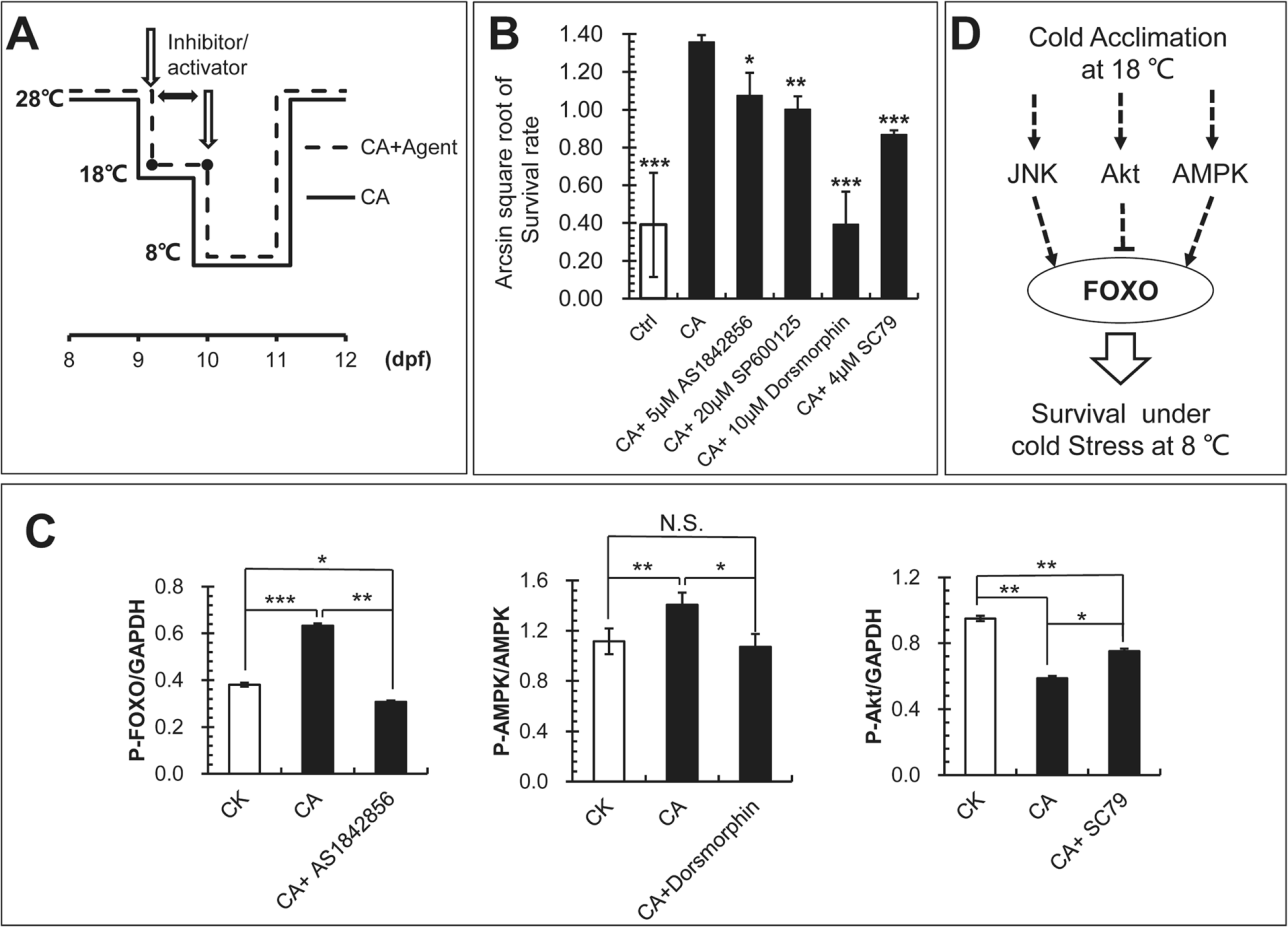
Verification of FoxO-related signaling pathways involved in cold acclimation of Songpu mirror carp. **a** Flow chart of these experiments. Larvae of Songpu mirror carp were pre-treated at 18 °C with or without inhibitors or activator for 24 h and then moved to severe low temperature at 8 °C for 24 h. Cold acclimation was monitored at 12 dpf by calculation of larval survival rates. **b** Cold acclimation of Songpu mirror carp was significantly affected by inhibition or activation of key signaling factors acting upstream of FoxO signaling. Larvae of Songpu mirror carp were treated with inhibitors (AS1842856 for FOXO at 5 μM, dorsomorphin 2HCl for AMPK at 10 μM or SP600125 for JNK at 20 μM) or an activator (SC79 for Akt at 4 μM) for 24 h. **c** The expression ratios of p-FOXO/GAPDH, p-AMPK/AMPK and p-Akt/GAPDH were calculated with the densitometries of Western blots measured using ImageJ software (NIH). Data of Western blots in Fig. S7 showed significant changes in phosphorylated protein levels after treatment with AS1842856 for FOXO at 5 μM, dorsomorphin 2HCl for AMPK at 10 μM or SC79 for Akt at 4 μM for 24 h. *, *p* < 0.05; **, *p* < 0.01; ***, *p* < 0.001. **d** A working model for the roles of FoxO-related signaling pathways in cold acclimation of carps

Next, inhibitors of FOXO (AS1842856), AMPK (Dorsomorphin 2HCl) and JNK (SP600125) and activator of Akt (SC79) were used to further verify the function of FoxO signaling in the formation of cold acclimation of Songpu mirror carp following a strategy in Fig. 7a. Briefly, a final concentration of AS1842856 at 5 μM, Dorsomorphin 2HCl at 10 μM, SP600125 at 20 μM or SC79 at 4 μM in the embryo medium was used to treat twenty 9-dpf larvae of Songpu mirror carp in 50-mm dishes during cold acclimation at 18 °C for 24 h. Then, the larvae were subjected to lethal cold stress at 8 °C for 24 h in the pre-cooled embryo medium without inhibitor or activator. Lethal cold-treated larvae were recovered at 28 °C for 24 h and the survival rates of larvae in different groups were calculated.

As shown in Fig. 7b, the survival rate of Songpu mirror carp decreased to 76.62, 70.9 and 16.72% after inhibition of FOXO, JNK and AMPK signaling pathways, respectively. Moreover, the survival rate of Songpu mirror carp was significantly reduced after activation of Akt signaling by SC79. We further detected the effects of inhibitors or activator on the phosphorylation levels of FOXO, AMPK and Akt using western blots (Figures S7, S8 and S9). In comparison with those in the control at 28 °C (CK), levels of phosphorylated FOXO and AMPK significantly increased but phosphorylated Akt decreased during cold acclimation at 18 °C (CA); however, treatments with inhibitors (AS1842856 for FOXO and Dorsomorphin 2HCl for AMPK) abolished cold-induced phosphorylation of FOXO and AMPK (Fig. 7c, left and middle panels). Additionally, the inhibition of Akt phosphorylation during cold acclimation (CA) was blocked by treatment with the SC79 activator (Fig. 7c, right panel).

Taken together, these data suggest that FoxO signaling played a crucial role in cold acclimation of two carp species and distinctive signaling pathways upstream of FOXO activation contributed to the difference of cold acclimation in Songpu mirror carp and Barbless carp.

## Discussion

Fish is the largest family of vertebrates containing about 33,100 species that are widely distributed around the world (https://www.fishbase.se/search.php). To cope with variable water temperatures, fish have evolved their features of thermal specialization and limitation through alterations at various levels from molecules and membranes to whole organisms and behaviors in ecosystems [42]. However, it remains largely unknown about biological processes and signaling pathways that are required for the development of difference in cold tolerance and cold acclimation of fish. Songpu mirror carp and Barbless carp are two closely related species of the carp family since they can hybrid to generate the offspring [39]. Previous studies have shown that Songpu mirror carp can easily survive in a frozen-over pond and some free water below the ice, but Barbless carp cannot survive in ponds of Heilongjiang province during winter [36]. In this study, we found that Songpu mirror carp exhibited stronger abilities of cold tolerance and acclimation than Barbless carp, which provided an ideal model to dissect molecular mechanisms underlying the development of cold tolerance and acclimation. We next performed a high-throughput RNA-seq analysis and identified many differentially expressed genes in Songpu mirror carp and Barbless carp during acute cold stress and acclimation. These differentially expressed genes were highly enriched in multiple biological processes and signaling pathways that are closely associated with cold tolerance and acclimation of two carp species. Moreover, FoxO-related signaling pathways appear to play crucial roles in the formation of strong cold tolerance and acclimation of Songpu mirror carp.

In addition to Songpu mirror carp and Barbless carp, organisms including zebrafish [33, 34], *Caenorhabditis elegans* [43, 44] and plants [45] possess a cold acclimation ability to allow their survival in extremely low temperature. In this study, many genes that are previously reported to associate with the cold acclimation of zebrafish [33] were found to be differentially expressed. For instance, *cyp7a1* (cholesterol 7-alpha-monooxygenase) is up-regulated by 2.7-fold in group II (b) and 3.5-fold in group III (b) during the formation of a strong cold acclimation in Songpu mirror carp, but the expression level of gene encoding Acyl-CoA desaturase is 2.3-fold lower in group II (b’) of Songpu mirror carp than that in Barbless carp (Fig. 3c) during acclimation (Additional file 5). Cholesterol 7-alpha-monooxygenase catalyzes the hydroxylation of carbon hydrogen bond at 7-alpha position of cholesterol and Acyl-CoA desaturase contributes to the biosynthesis of membrane phospholipids, cholesterol esters and triglycerides. Cholesterol content in animal cell membranes is negatively correlated to the fluidity of cell membrane and elimination of cholesterol from cells leads to the increment of membrane fluidity at low temperature [46]. Thus, the relatively low level of cholesterol content is likely associated with the strong cold acclimation of Songpu mirror carp.

Consistent with one of our previous studies during cold acclimation of zebrafish [33], we found the gene of *hmgb3* (high mobility group protein B3) and *cirbpb* (cold-inducible RNA-binding protein B) was up-regulated by 2.7- and 2.8- folds in Songpu mirror carp, and by 3.1- and 3.5-folds in Barbless carp during cold acclimation (Additional file 5). Another two genes (*cry2* and *nr1d2)* that are related to circadian clocks were also induced during cold acclimation of Songpu mirror carp and Barbless carp. These differentially expressed genes can be used as potential molecular markers for characterization of cold acclimation responses in different fish species.

Ca^2+^ is an important second messenger regulating various cellular processes and calcium homeostasis can be disrupted by cold atmospheric plasma [47]. The stenothermal fish burbot (*Lota lota L.*) can protect its heart from the cold by modification of calcium delivery system, which triggers the muscles contractions for a continue heart function at near freezing temperatures [48]. Ca^2+^ signaling also plays a crucial role in conferring cold tolerance in plants [45, 49, 50]. In this study, we found that the gene of *camk1g* encoding calcium/calmodulin-dependent protein kinase type 1G is up-regulated during cold acclimation (Additional file 5), suggesting the level of cellular Ca^2+^ is tightly associated with the cold acclimation of both Songpu mirror carp and Barbless carp.

Cold acclimation in plants is relevant to the changes of lipid membrane to protect the organisms against cold stress [51–53] and lipid plays a very important role in acclimation at low temperature in *Arabidopsis* [54] and *Capsicum annuum L*. [55]. In zebrafish, cold acclimation can alleviate lipid peroxidation damage at extremely low temperature [34] and lipid catabolism can enhance the resistance to acute cold stress in zebrafish [29]. Moreover, cold acclimation of carp from 30 °C to 10 °C caused a restructuring of liver microsomal phospholipids [56]. In this study, the representative of enriched GO terms in groups III and IV (d and e) include lipid homeostasis (GO:0055088), lipid transport (GO:0006869), and lipid metabolism (GO:0006629) (Figure S3), indicating that the changes in lipid homeostasis plays an important role in the cold acclimation of both Songpu mirror carp and Barbless carp.

We also found some biological processes that are specifically enriched during the cold acclimation of Songpu mirror carp. The most representative of enriched GO terms is cellular component assembly involved in morphogenesis (GO:0010927) in groups II and III (a, b and c), including tissue development (GO:0009888), actin filament-based process (GO:0030029), supramolecular fiber organization (GO:0097435), muscle structure development (GO:0061061), heart development (GO:0007507), striated muscle tissue development (GO:0014706), extracellular structure organization (GO:0043062), extracellular matrix organization (GO:0030198), and actomyosin structure organization (GO:0031032) (Fig. 4a and S1). These findings suggest that the formation of a strong cold acclimation in Songpu mirror carp is closely associated with changes in multiple tissues.

The most representative of biological processes that are inhibited by cold in Barbless carp is positive regulation of macroautophagy (GO:0016239) (Fig. 4b and S6). This biological process includes regulation of cell cycle (GO:0051726), response to cytokine (GO:0034097), positive regulation of catabolism (GO:0009896), cellular response to external stimulus (GO:0071496), cellular response to nutrient levels (GO:0031669), response to decreased oxygen levels (GO:0036293), and response to endoplasmic reticulum stress (GO:0034976). It is likely that the limitation of these biological processes is mainly contributed to the weak ability of cold acclimation in Barbless carp.

Signal pathways that were significantly activated during the cold acclimation of both Songpu mirror carp and Barbless carp include insulin signaling pathway, cholesterol metabolism, PPAR signaling pathway, and spliceosome (Fig. 5a). Consistently, lipid homeostasis, lipid transport and lipid metabolism were highly enriched during the cold acclimation of both Songpu mirror carp and Barbless carp (Fig. 4a). PPAR *pathway* can play an important role in regulating lipid metabolism in mature adipocytes by increasing fatty acid trapping [57]. Thermogenesis is activated during the cold acclimation of mice however, thermogenesis is inhibited in Barbless carp but not in the Songpu mirror carp during the cold acclimation (Fig. 5b). Further investigations are need to examine the contribution of PPAR signaling pathway and thermogenesis to a strong cold acclimation of Songpu mirror carp.

FOXO proteins are a family of transcription factors that can be inhibited by protein kinase B (PKB/Akt) [58]. In contrast, FOXO proteins are activated by the stress-activated c-Jun N-terminal kinase (JNK) and the energy-sensing AMP-activated protein kinase (AMPK), upon oxidative and nutrient stress stimuli [41]. In the study, we found that FoxO signaling pathway is highly enriched in groups II and III (a, b and c) (Fig. 5a). This signaling pathway can regulate the expression of genes in cellular physiological events including apoptosis, cell-cycle control, glucose metabolism, oxidative stress resistance [41]. Previous studies have shown that phosphatidylinositol 3′-kinase (PI3K)-Akt signaling pathway could be activated by many types of cellular stimuli or toxic insults and regulates fundamental cellular functions such as transcription, translation, proliferation, growth, and survival [59]. Additionally, AMPK is very important in regulating mitochondrial biogenesis, autophagy, and mitophagy, cell growth and proliferation [60] and mitochondria biogenesis helps offset the decreases in mitochondrial activity in cold environments, providing an additional pathway for lipid oxygen diffusion through lipids [61]. Therefore, it is likely that FoxO-related signaling pathways play a key role in cold acclimation of Songpu mirror carp. Indeed, we found that the survival rate of Songpu mirror carp decreased when the activity of FoxO signaling pathway was affected by two inhibitors or one activatior (Fig. 7c).

Mechanistically, the phosphorylation of AMPK will increase to activate downstream signaling pathways, leading to a new homeostasis of metabolism in cells and the synthesis of some new proteins for cold acclimation of carps. During cold acclimation, JNK was also activated to mediate the activation of FoxO signaling pathway, thus contributing to the protection of fish body under lethal cold stress. In contrast, cold acclimation inhibited the activation of AKT signaling followed by stimulation of the phosphorylation of FOXO protein (Fig. 7d and S7). It will be of great interest to further understand the regulation of FoxO signaling by AKT, JNK and AMPK signaling pathways during the cold acclimation of carps.

## Conclusions

In this study, Songpu mirror carp was found to have stronger abilities of cold tolerance and acclimation than Barbless carp. High-throughput RNA-seq analysis have identified many of differentially expressed genes, biological processes and signaling pathways between Songpu mirror carp and Barbless carp during cold acclimation. These differentially expressed genes were highly enriched in multiple biological processes and signaling pathways that are closely associated with cold tolerance and acclimation of two carp species. Moreover, FoxO-related signaling pathways appear to play crucial roles in the formation of strong cold tolerance and acclimation of Songpu mirror carp. These findings have provided novel clues for further investigation of molecular mechanisms underlying the tolerance and acclimation to cold stress in fish.

## Methods

### Experimental fish

Fertilized eggs of Songpu mirror carp and Barbless carp were obtained from Heilongjiang River Fishery Research Institute of Chinese Academy of Fishery Sciences. Fertilized eggs were maintained in plastic tanks containing aeration water at 28 °C and hatched under a photoperiod of 14 h light:10 h dark. All experiments began with larvae at 8 dpf (days post-fertilization). The larvae were randomly selected and transferred into 50 mm plastic dishes (20 larvae per dish) containing 8 ml of the embryo medium. Each experiment contains at least three independent biological replicates. The embryo medium is composed of 30% Danieau’s solution that contains 19.3 mM NaCl, 0.23 mM KCl, 0.13 mM MgSO_4_•7H_2_O, 0.2 mM Ca (NO_3_)_2_, and 1.67 mM HEPES at pH 7.2. After the study, fish were euthanised with 0.04% tricaine (Sigma-Aldrich, A5040-25G) in the embryo medium. Water temperature is precisely controlled with Immersion Circulators and Coolers (PC200 Immersion Circulators, Thermo Fisher Scientific).

### Chemicals, inhibitors and activators

Inhibitors AS1842856 (Catalog no. S8222), and dorsomorphin 2HCl (Catalog no. S7306), were obtained from Selleck chemicals. Inhibitor SP600125 (catalog no. HY-12041), and activator SC79 (Catalog no. HY-18749) were purchased from the medchemexpress. DMSO was obtained from Sigma-Aldrich. Stock solutions of all chemicals were freshly prepared in DMSO, and the final concentration of DMSO in exposure media did not exceed 0.5% (v/v).

### Cold tolerance and acclimation

To compare differences in cold tolerance and acclimation between Songpu mirror carp and Barbless carp, larvae at 8 dpf were divided into groups of acute stress (AS) and cold acclimation (CA). Larvae in AS group were maintained at 28 °C before 10 dpf and then exposed to lethal cold at 10 °C or extreme lethal cold at 8 °C for 24 h. Larvae in CA group were maintained at 28 °C before 9 dpf, subjected to mild low temperature at 18 °C for 24 h, and exposed to lethal cold at 10 °C or extreme lethal cold at 8 °C for another 24 h. Then, larvae at 11 dpf in both AS and CA groups were recovered at 28 °C for 24 h, followed by counting and removing the dead larvae until no dead larvae were observed. Larvae displaying no heart beating and no response to mechanical stimuli were identified as death [33, 34].

### Sample collection and RNA-seq analysis

Larvae at 8 dpf of Songpu mirror carp and Barbless carp were kept in dark throughout the experiment to avoid the influence of light exposure on gene expression [34]. Twenty larvae at 10 dpf of Songpu mirror carp and Barbless carp growing at 28 °C were sampled in triplets and served as controls (Ctrl). Twenty larvae at 9 dpf of Songpu mirror carp and Barbless carp growing at 28 °C were subjected to cold acclimation at 18 °C for 24 h, sampled in triplets at 10 dpf and served as cold acclimation groups (CA). Thus, a total of twelve samples including six control samples at 28 °C (Ctrl) and six samples of cold acclimation at 18 °C (CA) were collected for RNA extraction, preparation of RNA library and RNA-seq [33].

### Bioinformatics analysis

The raw reads were preprocessed to remove low-quality data (Q < 20) and ambiguous bases (N) from both ends of the reads using PRINSEQ (version 0.19.3) [62]. The cleaned and polished data for paired reads were extracted using Pairfq (version 0.14.4) [63]. These high-quality clean reads were then mapped to the reference genome (V2.0.CommonC) downloaded at carpbase (http://www.carpbase.org) using HISAT (Hierarchical indexing for spliced alignment of transcripts) (version 2.1.0) [64] to get the BAM formation of the aligned files. Then the counts of reads were summarized using read summarization program featureCounts [65] after using the Samtools set of the command line to convert the binary BAM files into SAM files. These counts were used for gene differential expression analyses using the Bioconductor DESeq2 package [66, 67]. The nucleic acid sequences of all the genes in the database were used to blast against the Swiss-Prot and TrEMBL (the Swiss Institute of Bioinformatics and the European Bioinformatics Institute) protein database to get the UniProt-accession. After obtaining the UniProt-accession of the genes, their KEGG Orthology ID and GO Orthology ID were obtained with the online tool bioDBnet (http://biodbnet.abcc.ncifcrf.gov) for enrichment analysis (Fig. 2a).

### Quantitative real-time PCR (qPCR)

The qPCR assays were performed to validate the RNA-seq data. Kit purchased from Fermentas was used to synthesize first-strand cDNA from total RNA samples without contamination of genomic DNA as described previously [34]. The PCR primers were designed using Primer Premier 6.0 software. Since the subunit S11 of the ribosomal gene *40S* was not found to be differentially expressed in RNA-seq data among samples, so it was selected as the reference gene for the normalization of gene expression as described previously [68]. qPCR data analysis was performed following the protocol of Hellemans et al. [69].

### Analysis of differentially expressed genes

Genes with a fold change ≥1.5 and a *q*-value ≤0.05 were considered to be differentially expressed. Differentially expressed genes were classified with Venn’s diagrams by online tools (https://bioinfogp.cnb.csic.es/tools/venny/index.html). KEGG (Kyoto Encyclopedia of Genes and Genomes) and GO (Gene Ontology) enrichment analysis were performed using TBtools software (https://github.com/CJ-Chen/TBtools). REVIGO tool (http://revigo.irb.hr/) [70] was used to cluster and prune GO terms on the basis of *p*-values obtained from TBtools.

### Western blots

Larvae at 9 dpf of Songpu mirror carp were treated with or without inhibitors or activators of FoxO signaling pathway during cold acclimation at 18 °C for 24 h, collected and lysed in cell lysis buffer for Western and IP (Beyotime, P0013J) containing 1% protease inhibitors (Protease Inhibitor Cocktail, Bimake) and 1% phosphatase inhibitors (Phosphatase Inhibitor Cocktail, Bimake). Larvae lysates were centrifuged at 12,000 g for 10 min at 4 °C. The supernatants were transferred to new centrifuge tubes and quantified using the BCA Protein Assay Kit (Beyotime, P0010). Boiled for 5 min at 100 °C in a 1 × loading buffer, 20 μg of total proteins were subjected to SDS-PAGE and electrotransferred to a PVDF membrane (Millipore).

The membranes were blocked with 5% (wt/vol) dried milk in 1 × Tris-buffered saline-tween (TBST) overnight at 4 °C, and incubated with the following dilutions of antibodies: rabbit monoclonal antibody for phospho-FOXO3a (Ser253), 1:1000 (Beyotime, AF1783); rabbit monoclonal antibody (40H9) for phospho-AMPKα (Thr172), 1:1000 (Cell Signaling Technology, 2535 T); rabbit monoclonal antibody for AMPKα (D5A2), 1:1000 (Cell Signaling Technology, 5831 T); rabbit monoclonal antibody for GAPDH (D16H11), 1:5000 (Cell Signaling Technology, 5174 T)]. Washed with TBST for 3 times (5 min for each time), membranes were probed with horseradish peroxidase (HRP)-conjugated goat anti-rabbit IgG secondary antibody at a 1:5000 dilution. Immobilon Western blot (WB) chemiluminescence HRP (Millipore) was used as the substrate to obtain signals using a Fujifilm LAS-4000 imaging system. The expression ratios of p-FOXO/GAPDH, p-AMPK/AMPK and p-Akt/GAPDH were calculated with the densitometries of Western blots measured using ImageJ software (NIH).

### Statistical analysis

Statistical analysis was performed using SPSS 19.0 or Microsoft Excel software for windows. The arcsine square root-transformed values for survival rates of carp larvae were statistically analyzed with the independent-samples t-test. The correlation of data between RNA-seq and qPCR was analyzed using the Spearman’s rho test.

## Supplementary information

**Additional file 1.** Survival rates of Songpu mirror carp and Barbless carp under cold stress.

**Additional file 2.** Primers used for qPCR.

**Additional file 3.** Validation of RNA-seq data by qPCR.

**Additional file 4.** Differentially expressed genes between two groups.

**Additional file 5.** Differentially expressed genes in each group obtained through Venn’s diagrams.

**Additional file 6.** Results of GO enrichment analysis for each group.

**Additional file 7.** Representative GO terms for biological processes in each group.

**Additional file 8.** Results of KEGG enrichment analysis in each group.

**Additional file 9 : Figure S1.** GO enrichment analysis of up-regulated genes in groups II and III (a, b and c). Highly similar GO terms are linked by edges in the graph, where the line width indicates the degree of similarity. Bubble size indicates the frequency of the GO term in the underlying GOA database. Bubble color indicates the *p*-value of GO enrichment results. The representative terms are showed in font words.

**Additional file 10 : Figure S2.** GO enrichment analysis of down-regulated genes in group II and III (a’, b’ and c’). Highly similar GO terms are linked by edges in the graph, where the line width indicates the degree of similarity. Bubble size indicates the frequency of the GO term in the underlying GOA database. Bubble color indicates the *p*-value of GO enrichment results. The representative terms are showed in font words.

**Additional file 11 : Figure S3.** GO enrichment analysis of up-regulated genes in group III and IV (d and e). Highly similar GO terms are linked by edges in the graph, where the line width indicates the degree of similarity. Bubble size indicates the frequency of the GO term in the underlying GOA database. Bubble color indicates the *p*-value of GO enrichment results. The representative terms are showed in font words.

**Additional file 12 : Figure S4.** GO enrichment analysis of down-regulated genes in group III and IV (d’ and e’). Highly similar GO terms are linked by edges in the graph, where the line width indicates the degree of similarity. Bubble size indicates the frequency of the GO term in the underlying GOA database. Bubble color indicates the *p*-value of GO enrichment results. The representative terms are showed in font words.

**Additional file 13 : Figure S5.** GO enrichment analysis of up-regulated genes in group IV (f and g). Highly similar GO terms are linked by edges in the graph, where the line width indicates the degree of similarity. Bubble size indicates the frequency of the GO term in the underlying GOA database. Bubble color indicates the *p*-value of GO enrichment results. The representatives are showed in font words.

**Additional file 14 : Figure S6.** GO enrichment analysis of down-regulated genes in group IV (f’ and g’). Highly similar GO terms are linked by edges in the graph, where the line width indicates the degree of similarity. Bubble size indicates the frequency of the GO term in the underlying GOA database. Bubble color indicates the *p*-value of GO enrichment results. The representatives are showed in font words.

**Additional file 15 : Figure S7.** Western blots to verify effects of inhibitors or activator on total and/or phosphorylated protein levels of corresponding signaling molecules including Akt and FOXO (A) and AMPK (B). Ctrl-control; CA-cold acclimation; Dor-treated with AMPK inhibitor dorsomorphin 2HCl at 10 μM for 24 h; SC79-treated with Akt activator SC79 at 4 μM for 24 h; AS-treated with FOXO inhibitor AS1842856 at 5 μM for 24 h. GAPDH serves as the loading control. Western blots were quantified with ImageJ software.

**Additional file 16 : Figure S8**. The full original uncropped images used for generation of Western blots in Fig. S7A. Ctrl: control; CA: cold acclimation; Dor: treated with AMPK inhibitor dorsomorphin 2HCl at 10 μM for 24 h; SC79: treated with Akt activator SC79 at 4 μM for 24 h; AS: treated with FOXO inhibitor AS1842856 at 5 μM for 24 h.

**Additional file 17 : Figure S9.** The original uncropped images for generation of Western blots in Fig. S7B. Ctrl: control; CA: cold acclimation; AICAR: treated with AMPK activator AICAR (acadesine) at 500 μM for 24 h; Dor: treated with AMPK inhibitor dorsomorphin 2HCl at 10 μM for 24 h.

## Data Availability

The sequencing data have been deposited in NCBI Sequence Read Archive (SRA, https://trace.ncbi.nlm.nih.gov/Traces/sra/?study=SRP223359) and the accession number is SRP223359.

## Abbreviations

AFP: Antifreeze protein
AFGP: Antifreeze glycoprotein
SERCA: Sarcoplasmic reticulum Ca^2+^-ATPase
UPR: Unfolded protein response
ER: Endoplasmic reticulum
Swiss-Prot and TrEMBL: the Swiss Institute of Bioinformatics and the European Bioinformatics Institute
GO: Gene ontology
KEGG: Kyoto encyclopedia of genes and genomes
REVIGO: Reduce visualize gene ontology
PCA: Principal component analysis
PPAR: Peroxisome proliferator-activated receptor
AMPK: AMP-activated protein kinase
PKB: Protein kinase B
FOXO: Forkhead box O
JNK: c-Jun N-terminal kinase
