# Supplementary figures and images for: Transcriptomic profiling revealed key signaling pathways for cold tolerance and acclimation of two carp species
Source: BMC Genomics. 2020 Aug 5;21:539. doi: 10.1186/s12864-020-06946-8 (PMC7430846; doi:10.1186/s12864-020-06946-8)

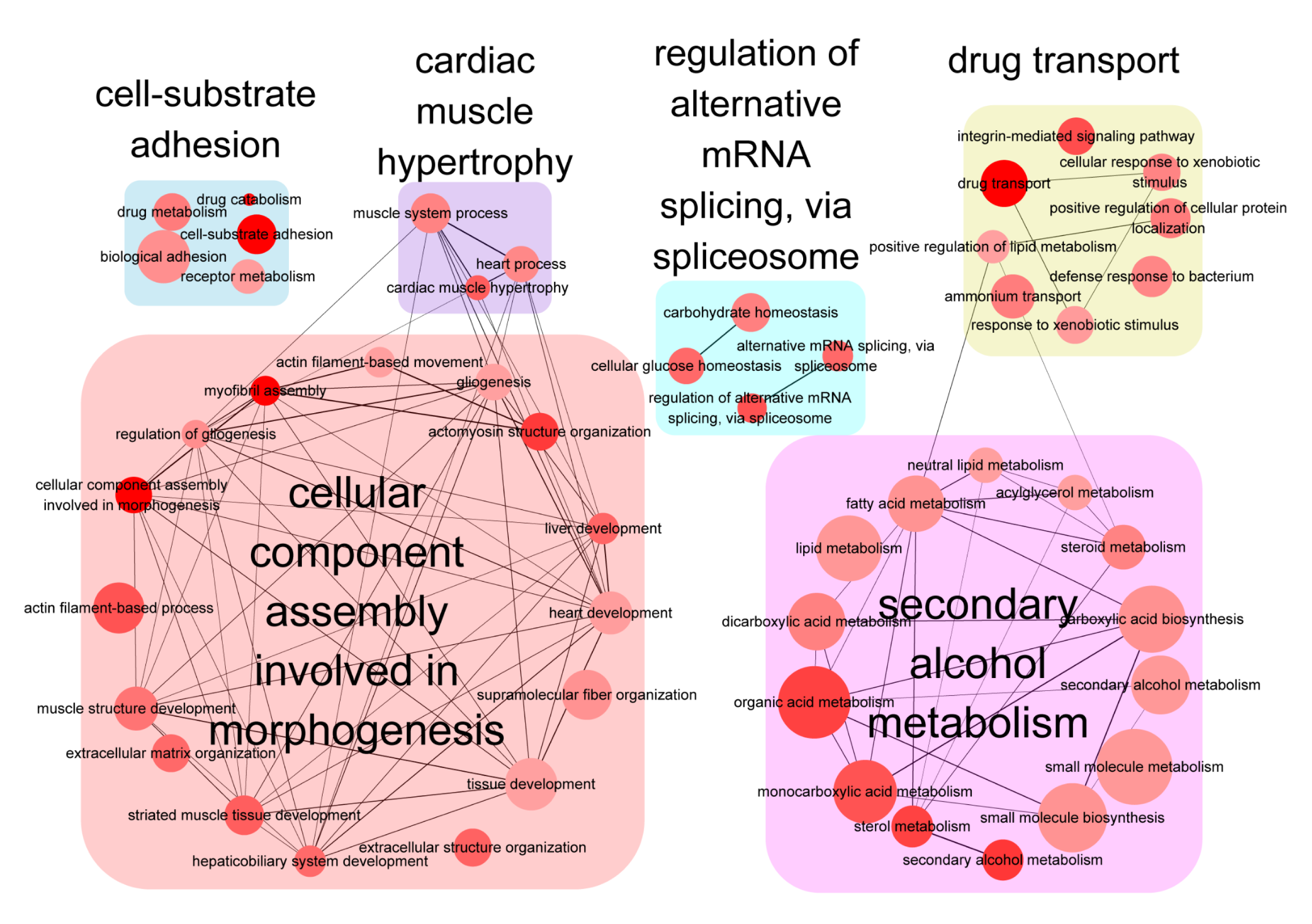

Supplement: Supplementary file 9 — Additional file 9 : Figure S1. GO enrichment analysis of up-regulated genes in groups II and III (a, b and c). Highly similar GO terms are linked by edges in the graph, where the line width indicates the degree of similarity. Bubble size indicates the frequency of the GO term in the underlying GOA database. Bubble color indicates the p-value of GO enrichment results. The representative terms are showed in font words. [file 12864_2020_6946_MOESM9_ESM.tif]

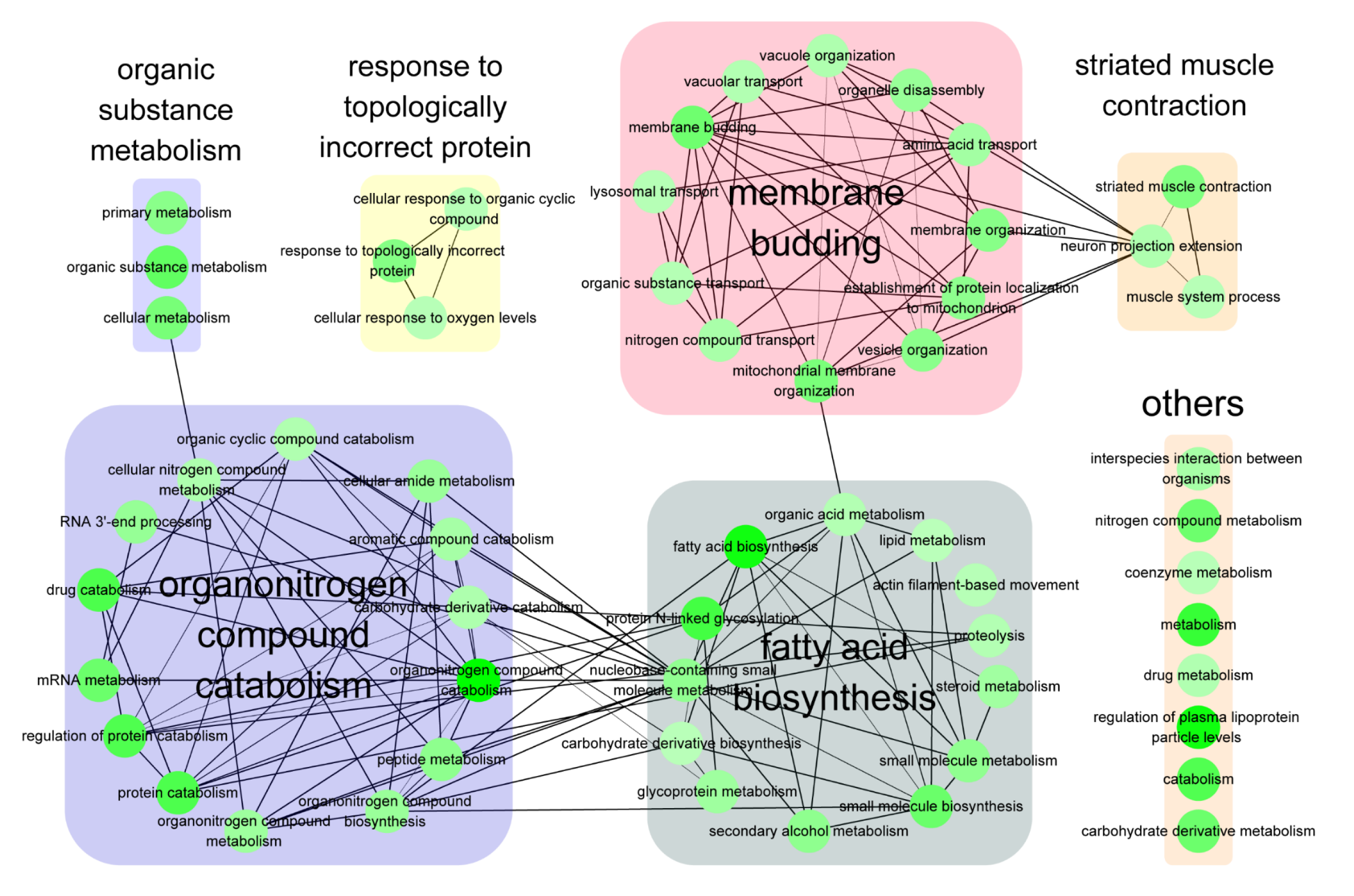

Supplement: Supplementary file 10 — Additional file 10 : Figure S2. GO enrichment analysis of down-regulated genes in group II and III (a’, b’ and c’). Highly similar GO terms are linked by edges in the graph, where the line width indicates the degree of similarity. Bubble size indicates the frequency of the GO term in the underlying GOA database. Bubble color indicates the p-value of GO enrichment results. The representative terms are showed in font words. [file 12864_2020_6946_MOESM10_ESM.tif]

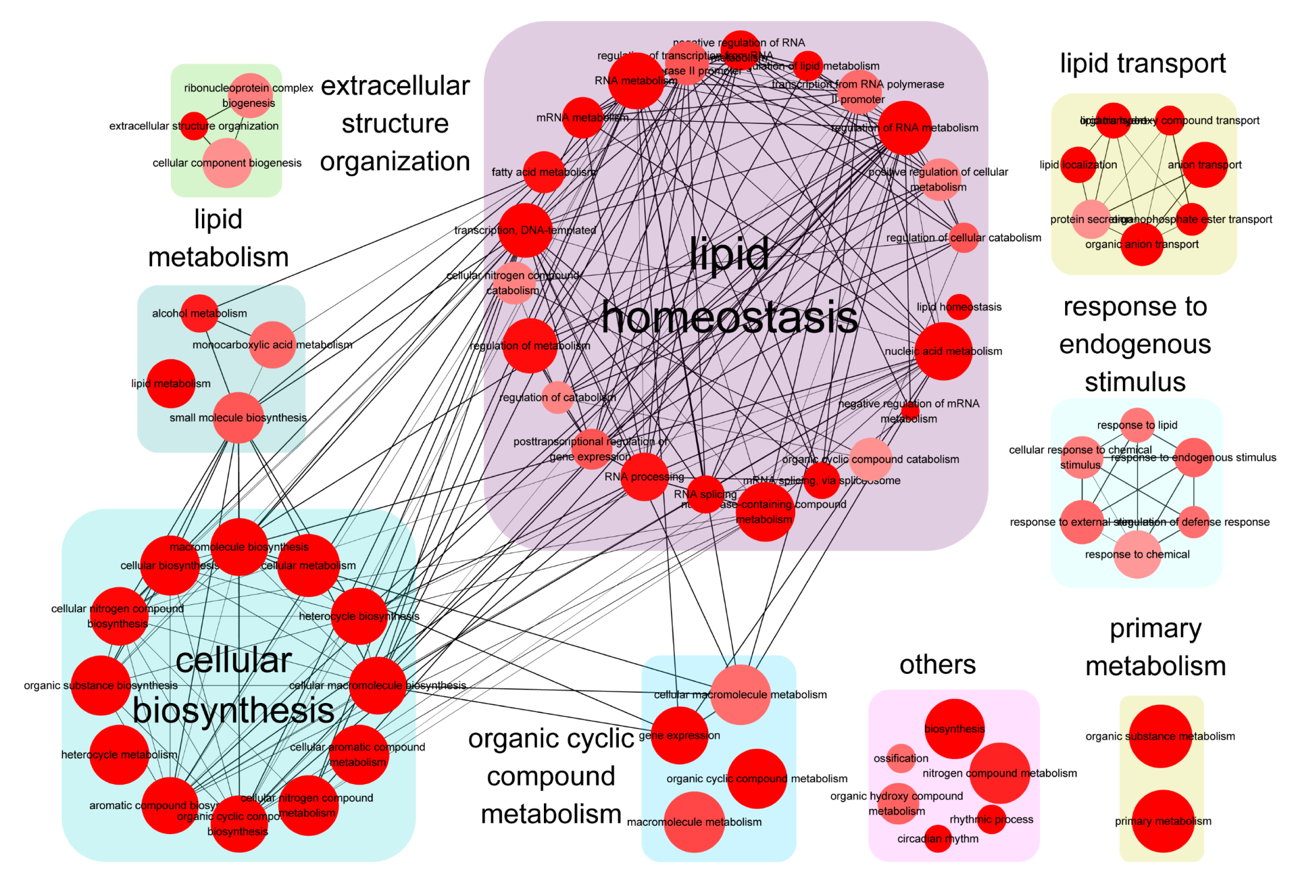

Supplement: Supplementary file 11 — Additional file 11 : Figure S3. GO enrichment analysis of up-regulated genes in group III and IV (d and e). Highly similar GO terms are linked by edges in the graph, where the line width indicates the degree of similarity. Bubble size indicates the frequency of the GO term in the underlying GOA database. Bubble color indicates the p-value of GO enrichment results. The representative terms are showed in font words. [file 12864_2020_6946_MOESM11_ESM.tif]

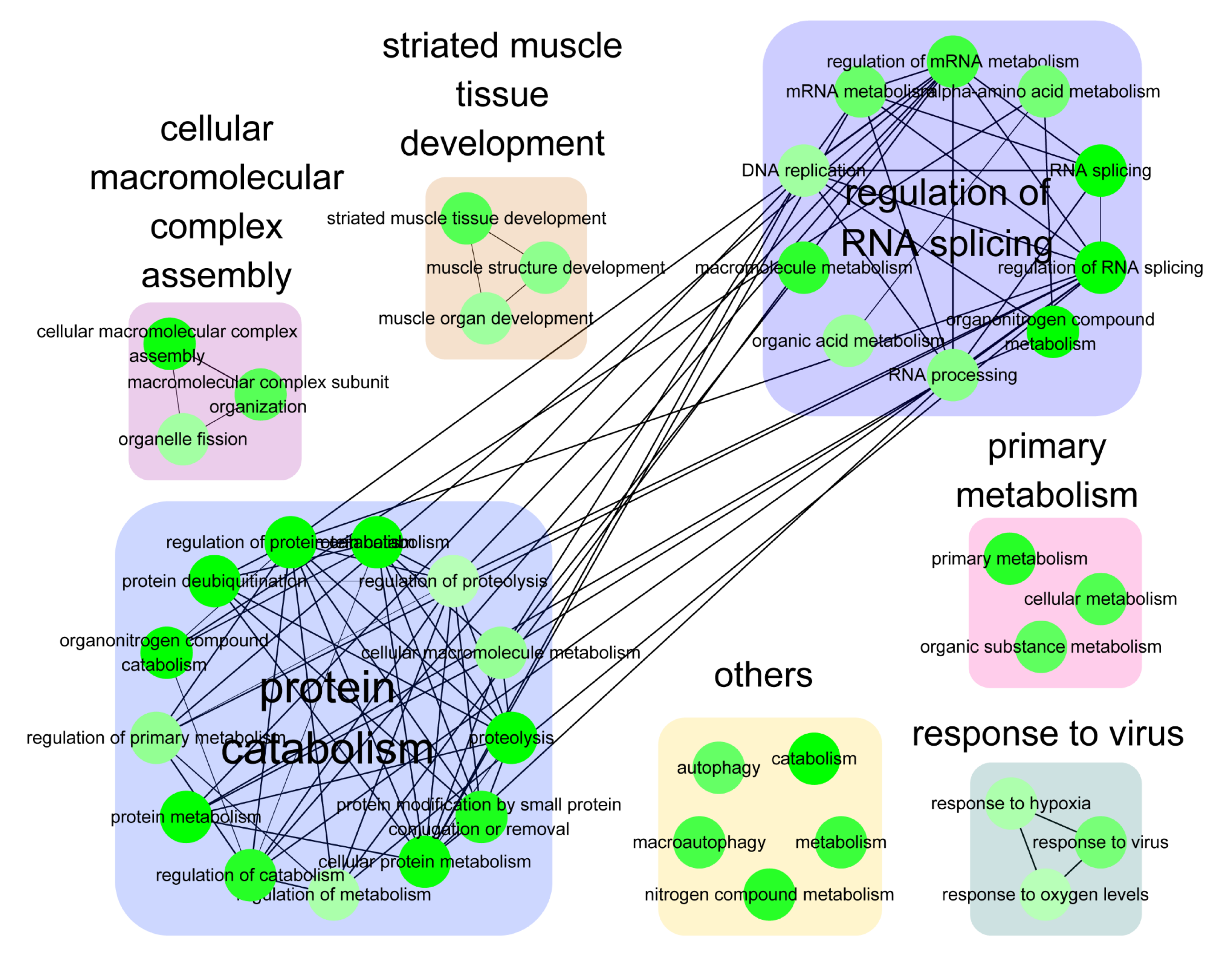

Supplement: Supplementary file 12 — Additional file 12 : Figure S4. GO enrichment analysis of down-regulated genes in group III and IV (d’ and e’). Highly similar GO terms are linked by edges in the graph, where the line width indicates the degree of similarity. Bubble size indicates the frequency of the GO term in the underlying GOA database. Bubble color indicates the p-value of GO enrichment results. The representative terms are showed in font words. [file 12864_2020_6946_MOESM12_ESM.tif]

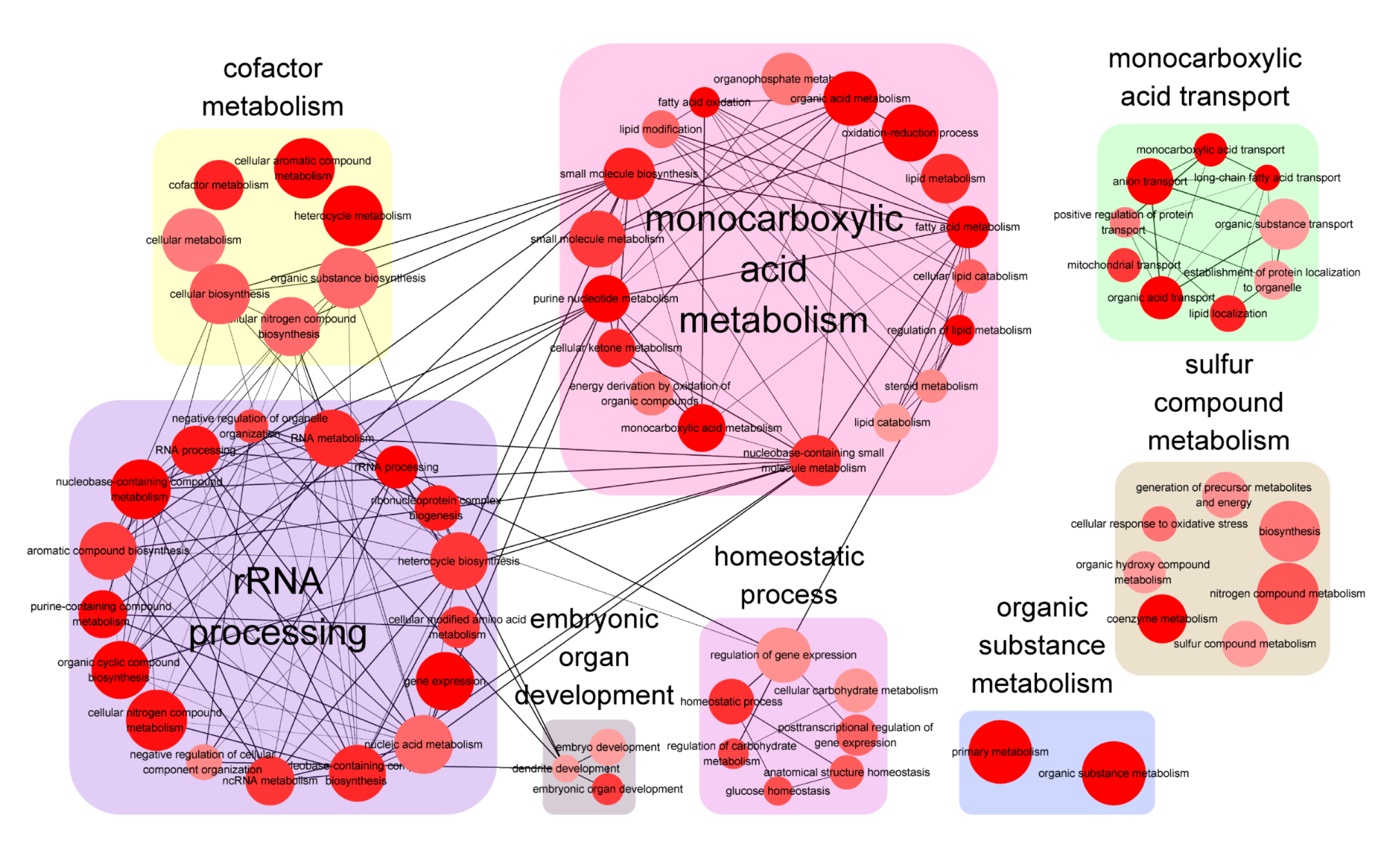

Supplement: Supplementary file 13 — Additional file 13 : Figure S5. GO enrichment analysis of up-regulated genes in group IV (f and g). Highly similar GO terms are linked by edges in the graph, where the line width indicates the degree of similarity. Bubble size indicates the frequency of the GO term in the underlying GOA database. Bubble color indicates the p-value of GO enrichment results. The representatives are showed in font words. [file 12864_2020_6946_MOESM13_ESM.tif]

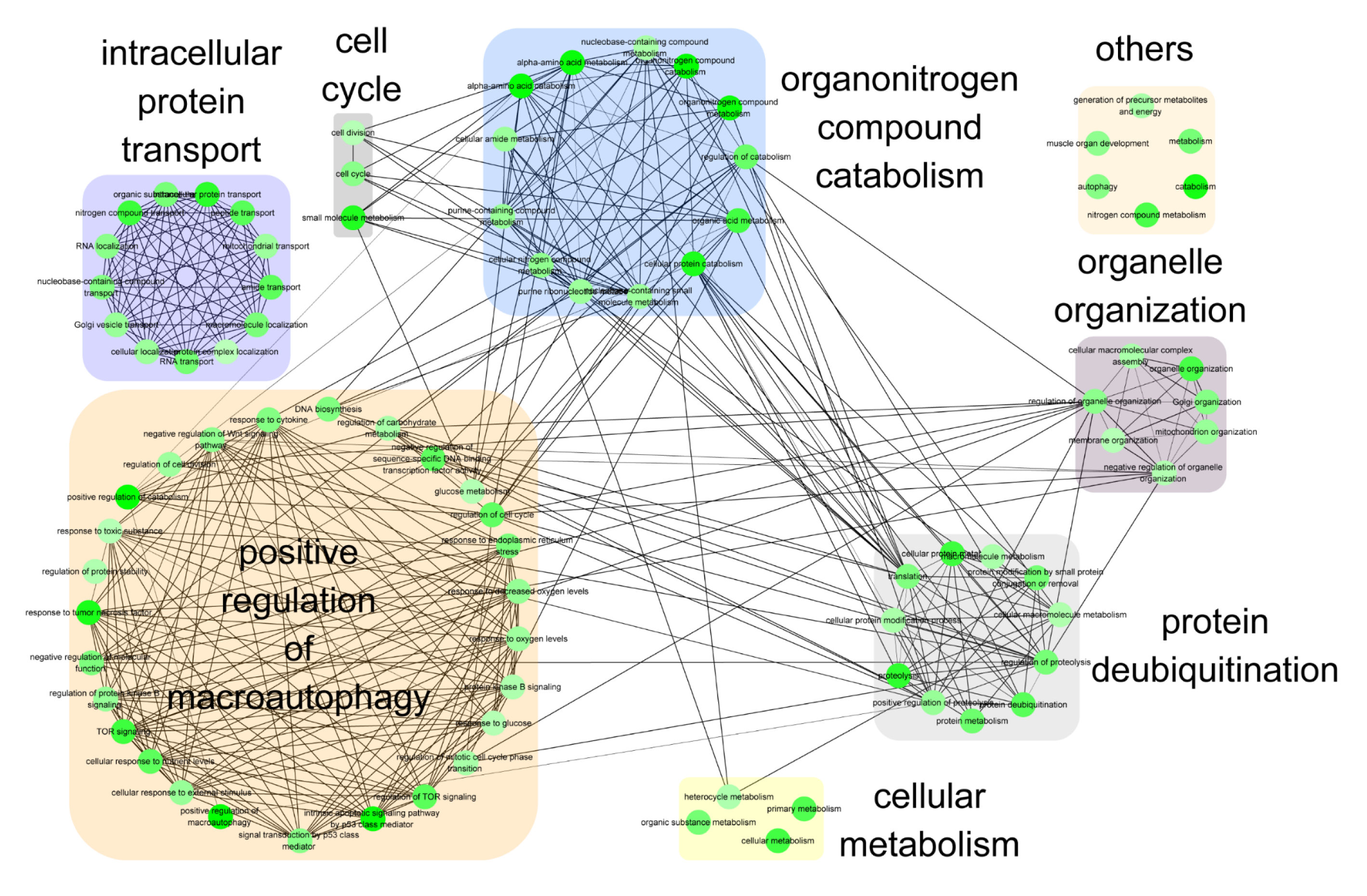

Supplement: Supplementary file 14 — Additional file 14 : Figure S6. GO enrichment analysis of down-regulated genes in group IV (f’ and g’). Highly similar GO terms are linked by edges in the graph, where the line width indicates the degree of similarity. Bubble size indicates the frequency of the GO term in the underlying GOA database. Bubble color indicates the p-value of GO enrichment results. The representatives are showed in font words. [file 12864_2020_6946_MOESM14_ESM.tif]

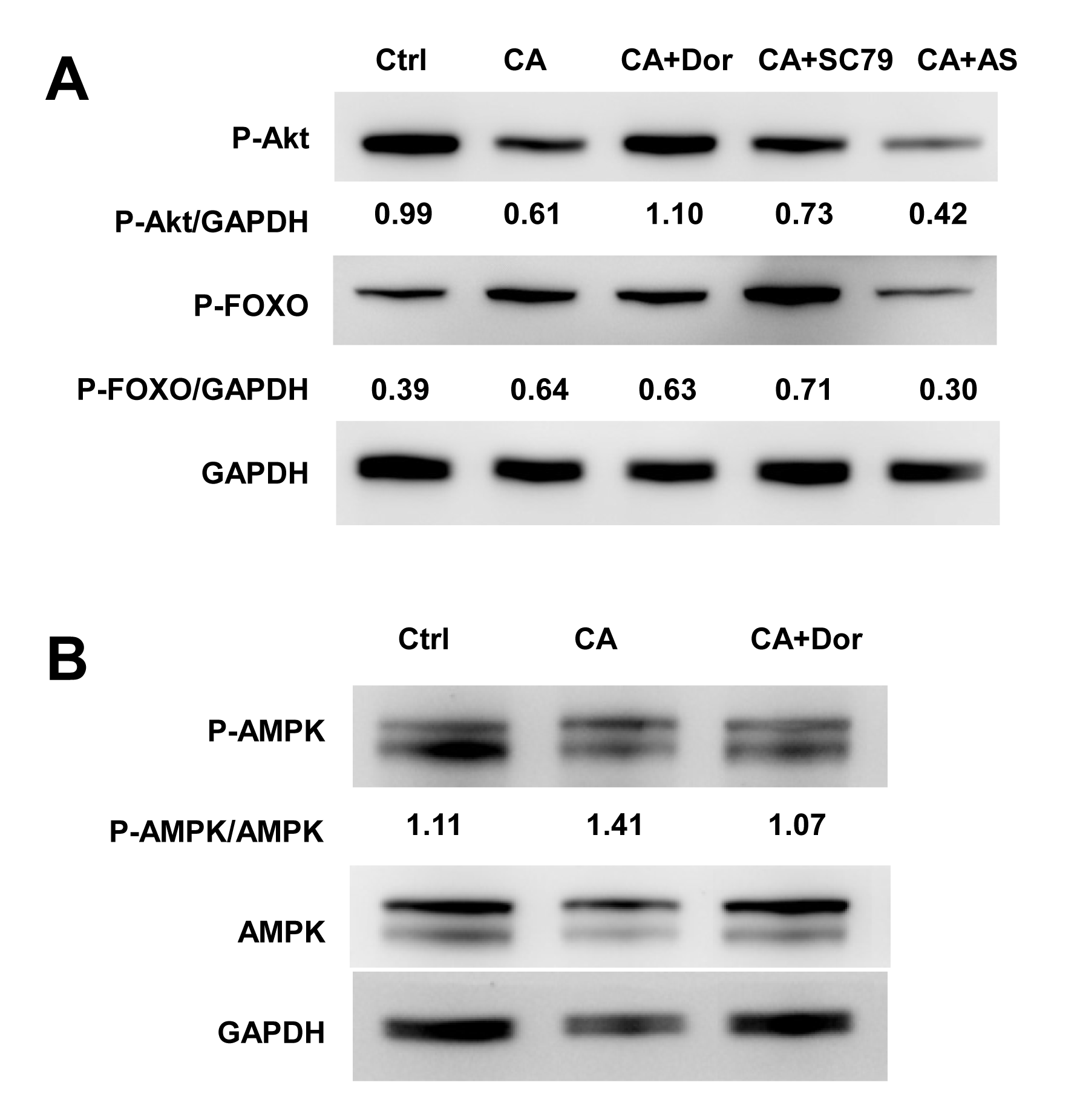

Supplement: Supplementary file 15 — Additional file 15 : Figure S7. Western blots to verify effects of inhibitors or activator on total and/or phosphorylated protein levels of corresponding signaling molecules including Akt and FOXO (A) and AMPK (B). Ctrl-control; CA-cold acclimation; Dor-treated with AMPK inhibitor dorsomorphin 2HCl at 10 μM for 24 h; SC79-treated with Akt activator SC79 at 4 μM for 24 h; AS-treated with FOXO inhibitor AS1842856 at 5 μM for 24 h. GAPDH serves as the loading control. Western blots were quantified with ImageJ software. [file 12864_2020_6946_MOESM15_ESM.tif]

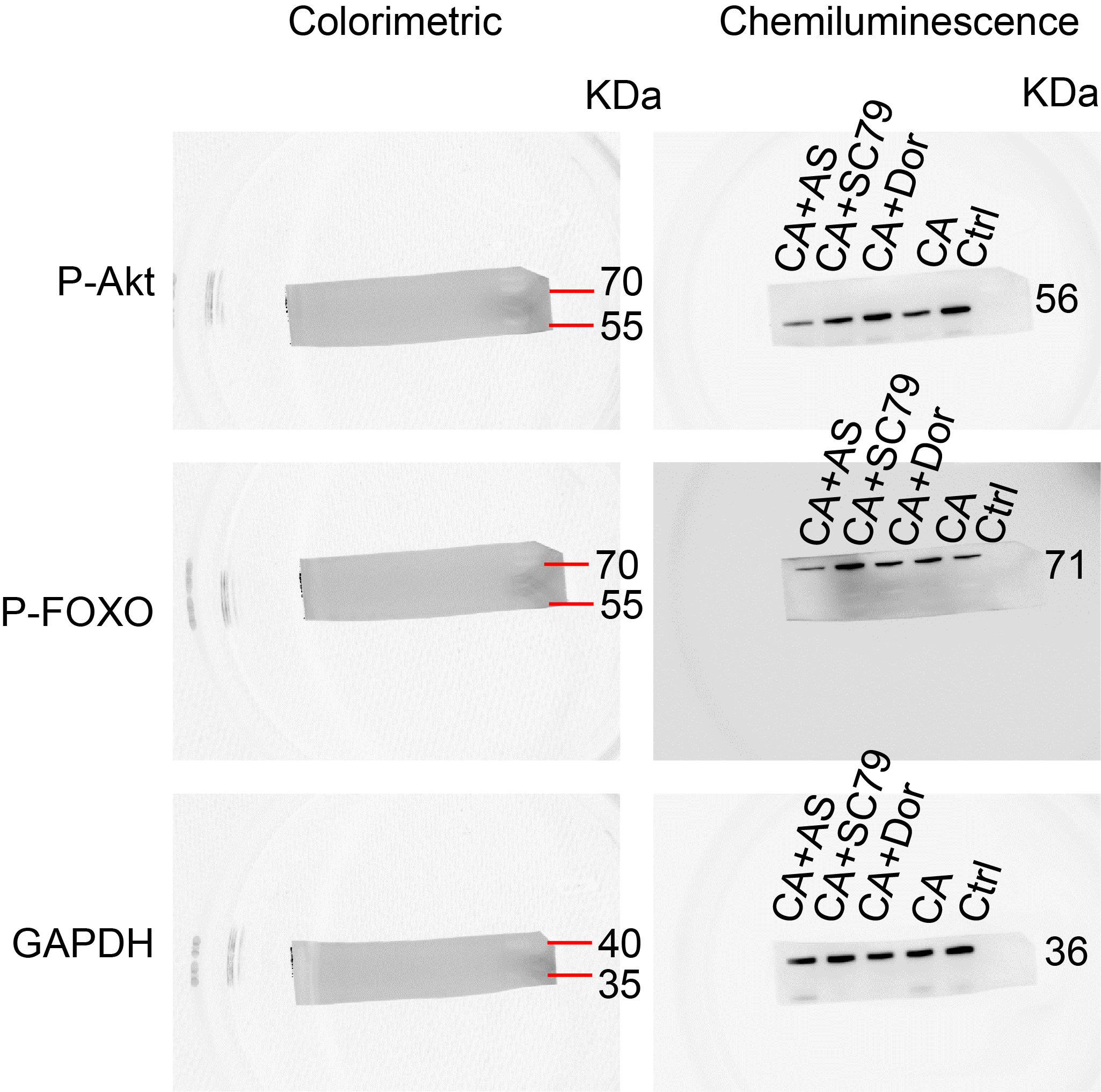

Supplement: Supplementary file 16 — Additional file 16 : Figure S8. The full original uncropped images used for generation of Western blots in Fig. S7A. Ctrl: control; CA: cold acclimation; Dor: treated with AMPK inhibitor dorsomorphin 2HCl at 10 μM for 24 h; SC79: treated with Akt activator SC79 at 4 μM for 24 h; AS: treated with FOXO inhibitor AS1842856 at 5 μM for 24 h. [file 12864_2020_6946_MOESM16_ESM.tif]

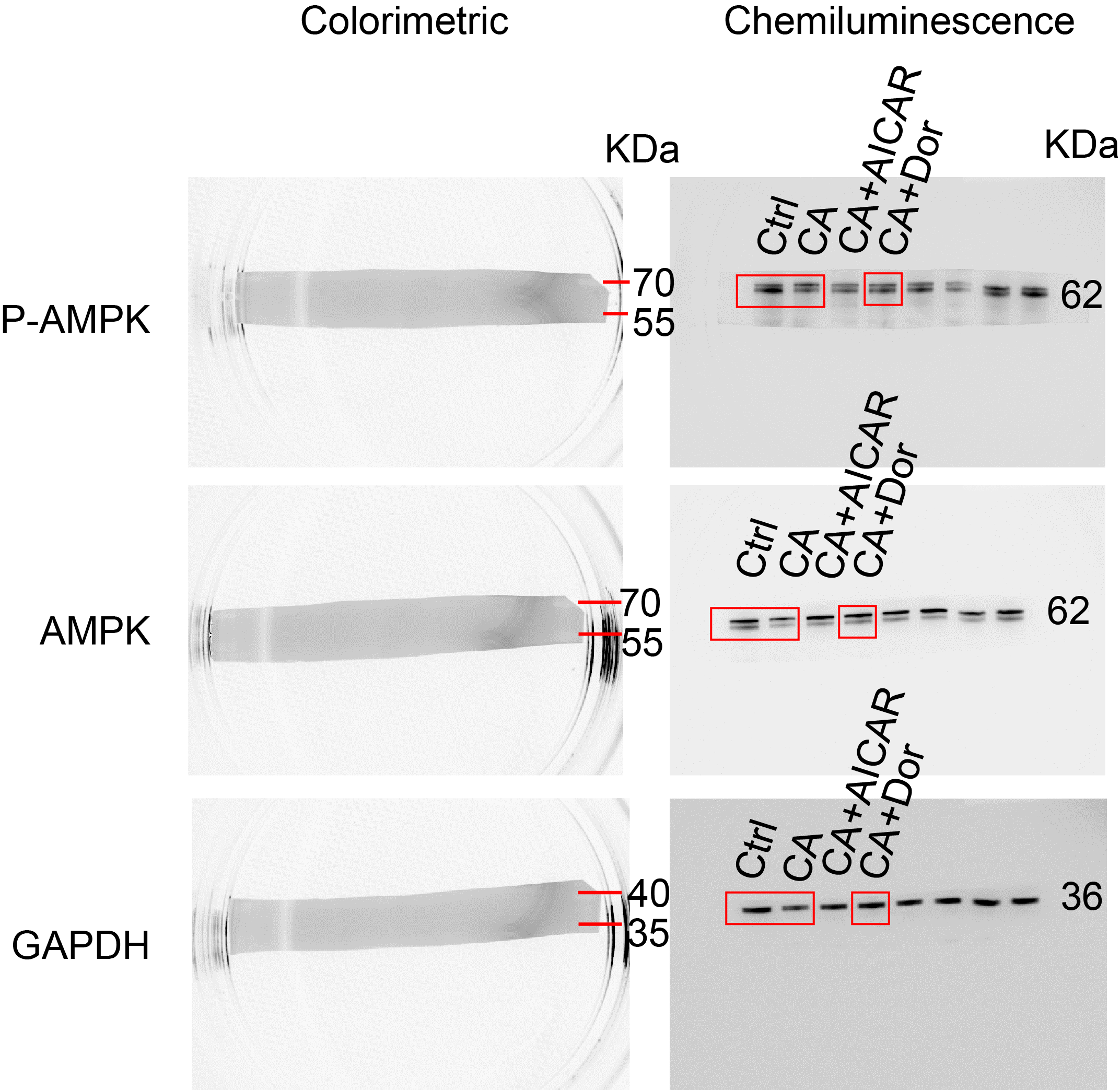

Supplement: Supplementary file 17 — Additional file 17 : Figure S9. The original uncropped images for generation of Western blots in Fig. S7B. Ctrl: control; CA: cold acclimation; AICAR: treated with AMPK activator AICAR (acadesine) at 500 μM for 24 h; Dor: treated with AMPK inhibitor dorsomorphin 2HCl at 10 μM for 24 h. [file 12864_2020_6946_MOESM17_ESM.tif]
